# A Transferable and Robust Computational Framework for Class A GPCR Activation Free Energies

**DOI:** 10.1021/acs.jpclett.5c03834

**Published:** 2026-03-03

**Authors:** Simone Aureli, Nicola Piasentin, Thorben Fröhlking, Valerio Rizzi, Francesco Luigi Gervasio

**Affiliations:** † School of Pharmaceutical Sciences, 27212 University of Geneva, Rue Michel-Servet 1, CH-1206 Geneva, CH, Switzerland; ‡ Institute of Pharmaceutical Sciences of Western Switzerland, 27212 University of Geneva, CH-1206, Geneva, CH, Switzerland; ¶ Swiss Bioinformatics Institute, 27212 University of Geneva, CH-1206, Geneva, CH, Switzerland; § Department of Chemistry, 4919 University College London, London WC1E 6BT, United Kingdom

## Abstract

The activation of G-protein coupled receptors is involved in many biomedically important cellular pathways. However, capturing it with molecular simulations is far from trivial, as it requires capturing both local and global motions. We recently achieved this goal in a specific receptor (the β1-adrenergic receptor, or ADRB1) by combining a multiple replica enhanced sampling approach with tailored collective variables. While that approach can be applied to other receptors, it would require a tedious and error-prone choice and refinement of the collective variables and, in particular, of the main path-like variable. Herein, we introduce an effective and streamlined evolved strategy for defining CVs that reduces user intervention while still achieving a robust free energy convergence. We apply it to two apo-GPCRs of pharmacological relevance, ADRB1 and the μ-opioid receptor. In the first case, we show that the reconstructed free energies agree with those obtained with the previous tailored approach, while for the μ-opioid receptor activation, we gain novel biological insights. The proposed method can be easily applied to other class A GPCRs, paving the way for the systematic elucidation of the activation mechanisms of many crucial drug targets.

## Graphical abstract

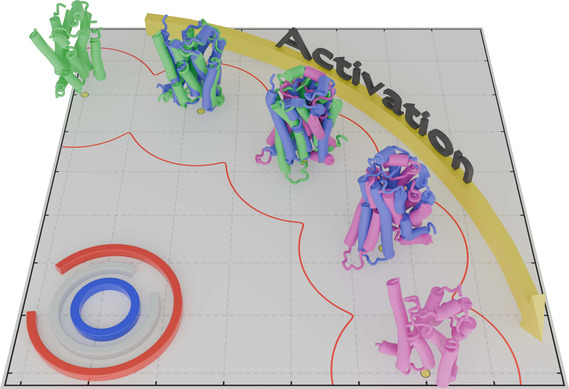

G-protein–coupled receptors (GPCRs) are one of the most important classes of membrane proteins, responsible for transducing extracellular signals into intracellular responses and serving as targets for a large fraction of approved drugs.^1–3^ Their function is tightly linked to conformational changes that span multiple scales, from local rearrangements of side chains and hydration sites to large-scale reorganizations of the transmembrane helices that define the transition between inactive and active states.^4,5^

Several simulation strategies have been applied to investigate GPCR activation, ranging from unbiased molecular dynamics (MD) simulations to various enhanced sampling methods.^6–17^ While unbiased MD simulations have revealed interesting details,^6–8,18,19^ receptors’ transitions occur on time scales that remain difficult to access with unbiased MD simulations, a limitation that is commonly addressed by enhanced sampling strategies.^12,14–16,20–23^ In particular, collective variable (CV)-based approaches aim to accelerate the reaction coordinates associated with the key processes under investigation.^24–27^

Among them, our carefully designed OneOPES framework enables simultaneous acceleration of multiple CVs, offering an efficient way to combine descriptors of both microscopic and mesoscopic motions within a single simulation.^28^ Recently, this strategy has been successfully applied to the activation of β1-adrenergic receptor (ADRB1), where specifically tailored CVs captured both local changes such as side-chain rearrangements and hydration dynamics, as well as the large-scale conformational reorganization of the receptor backbone.^17^

Within the OneOPES approach, the path collective variable (PATH CV, hereafter) has proven to be a natural and effective choice to represent GPCRs’ substantial backbone rearrangements. First introduced nearly 20 years ago,^29^ the original PATH CV requires the definition of a sequence of representative intermediate structures that bridge two target states, ideally arranged at regular intervals of RMSD. In practice, this setup can be labor-intensive; i.e., multiple manually curated structural hypotheses must be generated, tested, and refined in a trial-and-error process until a stable and physically meaningful path is obtained. This limitation reduces the accessibility and transferability of the method, making it challenging to apply it across multiple receptors or conformational transitions systematically.

Here, we present a robust and transferable strategy that streamlines the construction of PATH CVs for class A GPCRs, substantially reducing the level of manual intervention required while preserving the original accuracy. It is critical to emphasize that this approach requires only knowledge of the two target structures, eliminating the necessity to manually define and optimize a sequence of intermediate structures. We validate the approach on two pharmacologically relevant class A GPCRs in their apo forms, i.e., the β1-adrenergic receptor (ADRB1) and the μ-opioid receptor (MOR). For both systems, the refined PATH CV reproduces the free-energy landscapes obtained with the original tailored PATH CV approach but with shorter simulation times needed to achieve convergence and substantially reduced setup effort. This evolution thus improves both the efficiency and the transferability of the OneOPES sampling protocols for GPCR activation and, more broadly, for other biomolecular systems undergoing large-scale conformational transitions.

## Definition of the Euclidean PATH CV

The original RMSD-based PATH CV formulation,^29^ from now on referred to as RPATH, requires the definition of a sequence of representative intermediate conformations linking inactive and active states. This provides a *progress* variable *s* and a *deviation* variable *z* defined as follows:

1 $$\begin{array}{l} s\left(X\right)=\frac{\Sigma_{i=1}^{N}i\cdot e^{-\lambda R\left[X-X_{i}\right]}}{\Sigma_{i=1}^{N}e^{-\lambda R\left[X-X_{i}\right]}}\\ z\left(X\right)=-\frac{1}{\lambda }ln\left(\Sigma_{i=1}^{N}e^{-\lambda R\left[X-X_{i}\right]}\right) \end{array}$$

In this formulation, the path is described by a set of *N* reference structures or milestones *X*_*i*_, and the variables *s* and *z* are expressed as functions of the RMSD distances *R*[*X* – *X*_*i*_] between the current configuration *X* and each of these high-dimensional reference frames *X*_*i*_. The parameter λ determines the smoothness of the projection on the reference frames, with high λ values rigidly tying *s* to the closest single reference frame and producing a typical stepwise behavior in the dynamics, while low λ values spreading out *s* over a number of reference frames. In enhanced sampling, the sweet spot for λ tends to fall between these two regimes.

Building an RPATH CV requires a careful process of selecting the portion of the system undergoing the conformational change, and choosing and optimizing a number of reference structures. While structures of the end states are usually available, the intermediate structures have to be generated. One typically extrapolates them from biased simulations, such as steered MD or adiabatic bias MD^30,31^ (see Figure 1A). These explorative simulations accelerate a reaction coordinate to quickly sample transitions connecting the end states so that a trial sequence of conformations can be extracted from them. However, for large and complex biomolecular systems such as GPCRs, this process is particularly challenging and time-consuming, as it is difficult to both figure out a good reaction coordinate and to evaluate the quality of the extracted structures *a priori*. Eventually, preliminary biased simulations of the trial RPATH CV must be run to check whether the selected intermediates are sufficient to drive the desired conformational change. Numerous trial-and-error attempts are required before an effective CV is obtained. This recursive procedure is typically not transferable even between analogous systems, and the CV refinement process must be repeated for each new system under investigation.

**Figure 1:**
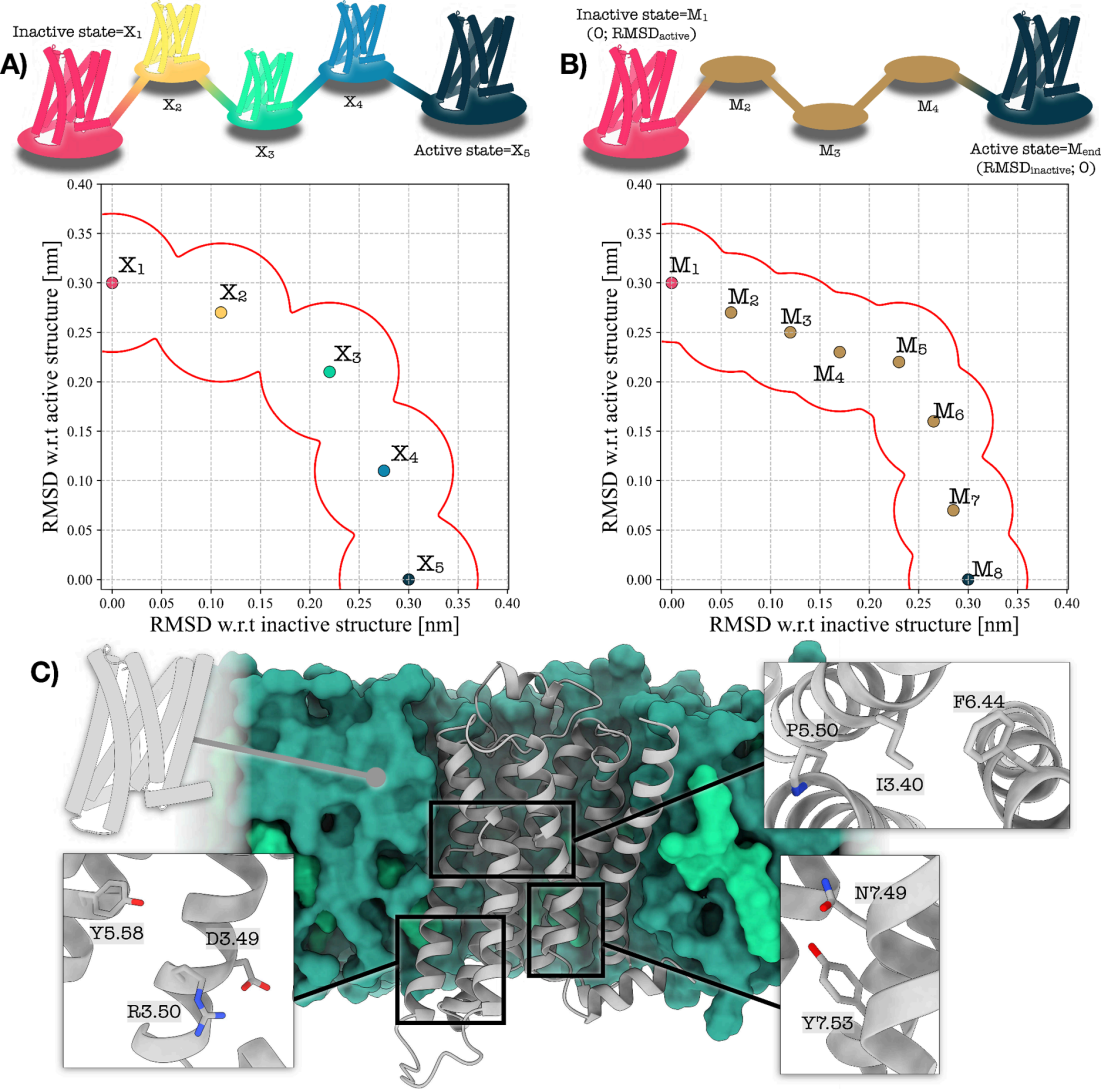
Schematic comparison between the original PATH CV^29^ and the Euclidean variant used in this work. A) In the traditional formulation, constructing a meaningful PATH CV for GPCR activation requires multiple intermediate PDB structures *X*_*i*_ along a putative transition pathway. Because these intermediates must be guessed and manually curated, the resulting path is laborious to set up and often uncertain in its direction, leading to repeated trial-and-error attempts. B) In the Euclidean variant, only the inactive and active structures are required, while all intermediate milestones are simply defined in terms of the two-dimensional space of RMSD values relative to these end points. This yields a path that is straightforward to define as it does not depend on any intermediate structure. C) Depiction of a prototypical class A GPCR (ADRB1) embedded into a model membrane. POPC and cholesterol molecules are displayed in dark and light green, respectively. Insets show the most relevant microswitches governing GPCR’s activation.

A further limitation of RPATH CV is the requirement to focus the RMSD calculation on specific portions of the GPCR. Typically one has to align the RMSD over the rigid portion of the GPCR and calculate the RMSD over the structural sections that change the most during the activation. Different alignment strategies on different sets of atoms yield vastly different RMSD measurements, critically impacting the quality of the resulting RPATH CV. This step as well requires a recursive optimization process that further slows down the overall procedure. Although reformulating the path in terms of contact maps has been shown to mitigate this problem by avoiding explicit structural superposition,^32–34^ a contact-map-based path CV remains difficult to build and cannot be transferred between systems.

A different and more straightforward approach was proposed to simulate the activation of the glucagon receptor.^16^ The RMSDs over the two inactive and active end states were calculated and transformed into a CV by simply calculating their difference. The resulting CV captures by construction the progress along the GPCR activation and is equivalent to a linear path between two milestones. However, its lack of curvature represents a limit, as it may force the system to pass through improbable high-energy states where both RMSDs are equally low. In the meantime, an alternative Euclidean formulation of the PATH CV, hereafter referred to as EPATH, was proposed and mostly used in the context of chemistry and catalysis.^35–38^ In this variant, the path is not defined by the RMSD value with respect to a sequence of structures but as progress in CV space. In other words, in EPATH the milestones *M*_*i*_ are represented by tuples of CV values and do not need to be tied to real structures.

Here, we introduce a strategy that puts together these two approaches: we propose an EPATH CV on a two-dimensional space that uses the two RMSD values with respect to a GPCR’s inactive and active structures. A crucial component of our strategy is the availability of high resolution structures of the end states over which we measure the RMSD. Along the resulting EPATH CV, any visited structure of the system is encoded in a tuple *M* defined by a couple of RMSD values (*RMSD*_inactive_(*M*), *RMSD*_active_(*M*)). In this way, only the end state milestones, i.e., *M*_1_ and *M*_8_ in Figure 1, have to be tied to real structures, while the milestones *M*_*i*_ in between can be generated arbitrarily to chart a route in RMSD space. Here, we choose a slightly arched path above the diagonal that retraces the shape of the path that we observed in our previous work on GPCR activation.^17^ This path connects the well-defined end states by passing through an entropic basin where both RMSD values are high at the same time, that is, a basin populated by states that differ significantly from both the inactive and active references.

Within the EPATH CV approach, the Euclidean distance between the milestone value *M* of a state and any reference milestone *M*_*i*_ is then simply defined as

$$d\left(M,M_{i}\right)=[{\left(RMSD_{inactive}\left(M\right)-RMSD_{inactive}\left(M_{i}\right)\right)}^{2}+{{\left(RMSD_{active}\left(M_{i}\right)-RMSD_{active}\left(M_{i}\right)\right)}^{2}]}^{1/2}$$

Substituting this squared distance into the expressions in eq 1 for *s*(*X*) and *z*(*X*) simply yields the EPATH CV value for structure *M* in a path made of *N* milestones:

2 $$\begin{array}{l} s\left(M\right)=\frac{\Sigma_{i=1}^{N}i\cdot e^{-\lambda d{\left(M,M_{i}\right)}^{2}}}{\Sigma_{i=1}^{N}e^{-\lambda d{\left(M,M_{i}\right)}^{2}}}\\ z\left(M\right)=-\frac{1}{\lambda }\ln\left[\Sigma_{i=1}^{N}e^{-\lambda d{\left(M,M_{i}\right)}^{2}}\right] \end{array}$$

This approach retains the definitions of progress (*s*(*M*)) and deviation (*z*(*M*)) from those of the original RPATH CV approach. Its key advantage is that it avoids the need to define intermediate milestones tied to a curated set of conformations. Consequently, setting up the resulting EPATH CV is much simpler, as the time-consuming recursive refinement procedure of the previous approach is not required. Another advantage of this strategy is that it relies less on RMSD alignment and the selection of atoms used to calculate it. To highlight this, we choose to align and calculate the RMSD over the same set of Cα atoms that make up the secondary structure of the system (more than 90% of the system’s atoms), and this generic choice had no need for refinement for building an efficient CV.

Finally, in terms of performance, the EPATH CV is faster to compute since the numerically expensive RMSD calculation needs to be carried out only twice per time step. Conversely, in the RPATH CV such calculation is computed *N* times per time step, once for each intermediate structural milestone. These advantages make applying the EPATH strategy to multiple systems more straightforward, reducing setup time, and improving transferability.

## OneOPES MD Simulations

The equilibrated structure of ADRB1 was taken from our previous work,^17^ where we generated it starting from the antagonist-bound inactive structure within PDB ID: 7BVQ.^39^ The structure of MOR was obtained from the inactive MOR structure within PDB ID: 9MQJ by removing the ligand resolved in the orthosteric binding site.^40^ The reference active structures of ADRB1 and MOR were retrieved from PDB ID: 7BTS^39^ and PDB ID: 8F7R,^41^ respectively. The details of the preparation and equilibration of MOR and ADRB1 are detailed in the Supporting Information and in Figure S1.

We used OneOPES,^28^ a replica-exchange extension of OPES Explore,^42^ to sample the activation pathway of two GPCRs, ADRB1 and MOR. In each simulation, eight replicas were run, i.e., one convergence-focused (replica **0**) and seven exploratory ones (replicas **1–7**) where additional CVs were accelerated. All replicas shared OPES Explore as the main sampling bias, while replicas **1–7** were progressively heated up to 335 K by biasing the system’s potential energy with OPES Expanded^43^ to facilitate barrier crossing.

To directly compare the two approaches, we ran simulations using the GPCR activation PATH CV in both formulations, i.e., the original RPATH^29^ and the newer EPATH implementation. In analogy with ref 17, additional CVs were introduced for four key microswitch motifs,^44^ i.e., *PIF*, *DRY*, *NPxxY*, and *YY*, to capture local structural determinants of activation. The distances within these motifs were biased as auxiliary CVs in the exploratory replicas. Both approaches use a similar set of parabolic restraints on the microswitches PIF and NPxxY to guide the system through the intermediate basins, which contain many metastable states and kinetic traps, to the fully active one. It is important to highlight that these parabolic restraints only apply to intermediates and not to end-point states, and as such they do not affect their relative stability.

In addition, we biased the hydration of the cytoplasmic cavity, as our recent studies have shown that water coordination at specific sites can be critical for lowering the activation free-energy barrier.^45–51^ In the case of ADRB1, this choice is further supported by NMR evidence indicating that the *YY*-motif is stabilized through a water-mediated interaction.^52^

For analyzing and comparing results, directly using the biased RPATH and EPATH CVs would be rather ineffective. We chose instead to reproject the free energy results on an alternative simplified EPATH CV with fewer milestones, six for ADRB1 and seven for MOR. Further analyses and figures on the biased PATH CVs are instead reported in the Supporting Information.

Simulations were performed using GROMACS 2023 ^53^ patched with PLUMED 2.9.1.^54,55^ Full details about simulation parameters and CVs are reported in the Supporting Information, respectively in Tables S1 and S2 and Figures S2 and S3, and the simulation input files are provided in GitHub at https://github.com/valeriorizzi/GPCR_Euclidean_PATH.git.

## Cluster Analysis and Shannon’s Entropy

We performed a cluster analysis on the MOR’s OneOPES simulations using GROMACS’s *gmx cluster* routine, using the *gromos* algorithm. An RMSD threshold value of 1.4 Å on the secondary structure Cαs was selected (the same selection of Cαs we used for RMSD_*inactive*_ and RMSD_*active*_), considering the number of generated cluster families and the similarity of protein conformations within a cluster family. From the cluster population, the Shannon’s entropy was calculated, using the following formula:

3 $$S=-k_{B}\overset{C}{\underset{i=1}{\sum }}p_{i}\ln\left(p_{i}\right)$$

where *k*_B_ is the Boltzmann constant, *p*_*i*_ is the fractional population of the *i*-th cluster with respect to all the cluster families *C* and $\overset{C}{\underset{i=1}{\sum }}p_{i}=1$ . In the analysis, once we partition the phase space into different regions, we use this metric as a natural measure of configurational diversity in each region. Regions with one dominant cluster family would display low entropy values, whereas regions with numerous equally probable cluster families would have high entropy content.

## Validation of the Euclidean PATH CV on ADRB1

We first assessed the performance of the EPATH CV on ADRB1, a prototypical class A GPCR that has been widely investigated both experimentally and computationally.^39,52,56,57^ Using the same simulation setup and CV selection of our previous study,^17^ we repeated OneOPES simulations of the apo-ADRB1 system, replacing the RPATH CV with the newly constructed one (see Figure 2A and Figure S4A). The resulting free-energy landscape along the activation pathway closely matches our earlier results, recovering both the relative stability of the inactive and active basins and the barrier separating them (Figure 2B). Notably, the simulations converge in significantly shorter time scales than with the RPATH CV, highlighting the efficiency gain of the EPATH approach without compromising accuracy (see Figure 2C and Figure S4B,C).

**Figure 2:**
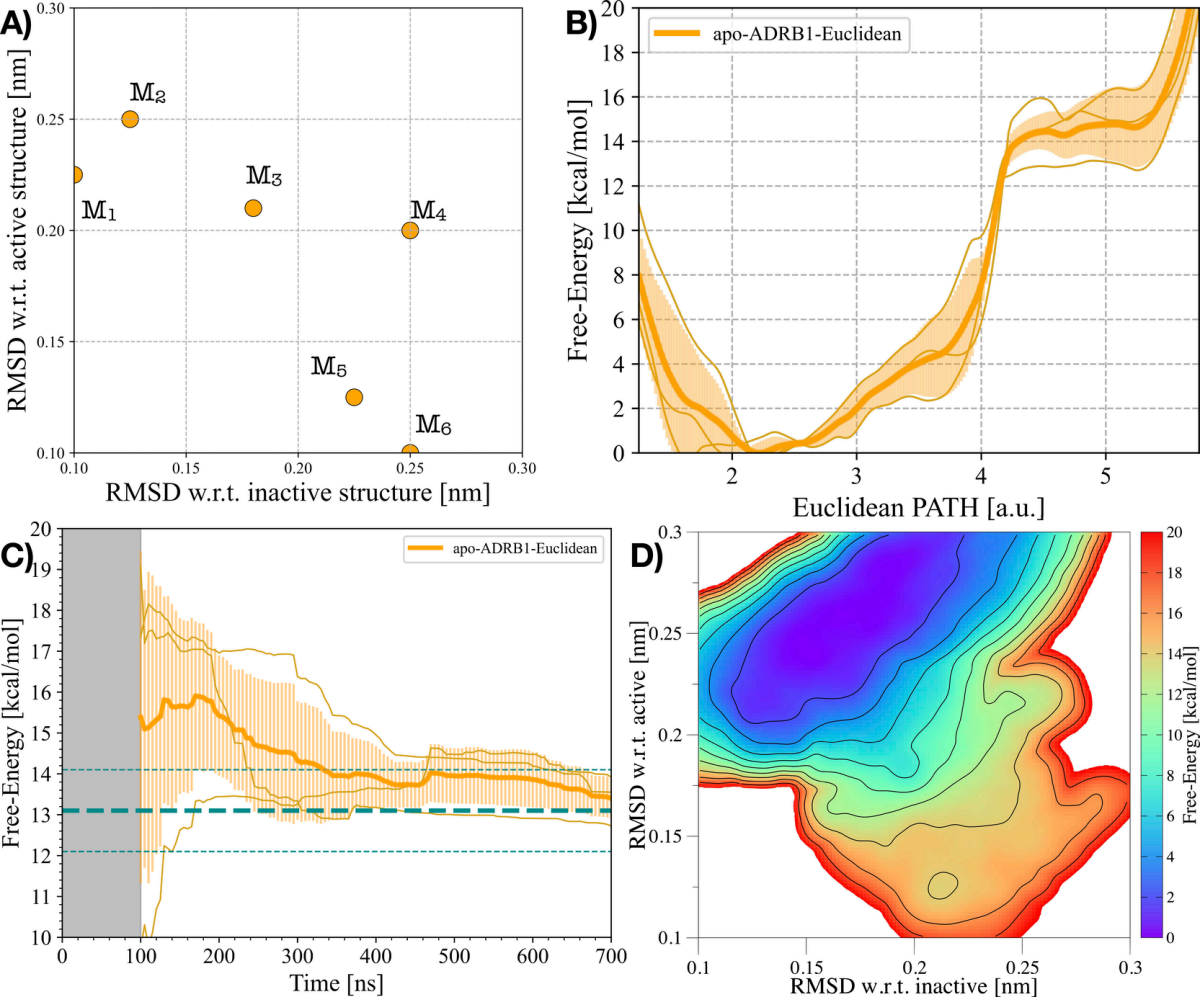
ADRB1’s activation using the EPATH CV. A) Milestones M_*i*_ of the EPATH CV employed in *apo-ADRB1-Euclidean* and in ref 17. B) 1D FES as a function of the EPATH CV in (A) for the *apo-ADRB1-Euclidean* system. C) Free-energy difference between inactive and active states in the *apo-ADRB1-Euclidean* system over time. In (B) and (C), the average data over three replicas are represented through an orange solid line, while the transparent orange area illustrates the standard deviation. D) Average 2D FES concerning the RMSD of both the inactive and active structures for the *apo-ADRB1-Euclidean* system.

As a further form of comparison, we reprojected the accumulated bias potential onto the (*RMSD*_inactive_; *RMSD*_active_) plane, obtaining a two-dimensional free-energy surface (FES) describing the conformational change leading to ADRB1’s activation (Figure 2D). This representation confirms that the 2D FES obtained with the EPATH is in excellent agreement with the one previously computed using the RPATH, with both inactive- and active-like basins recovered at the expected locations. Additional analyses of cavity hydration and the conformational rearrangements of key microswitches during ADRB1 activation are provided in the Supporting Information (see Figure S5) and show a good quality agreement between the two approaches.

## Activation of the Mu Opioid Receptor

In this section, we report the simulation results of the activation of another class A GPCR: the μ-opioid receptor (MOR). MOR is one of the most critical analgesic targets and is at the center of the ongoing opioid crisis.^58,59^ We ran OneOPES with both the RPATH and EPATH CV definitions. The former was extrapolated from a series of steered MD between representative inactive and active structures of MOR (for further details, refer to the Supporting Information and Figure S6), while the latter was defined through (*RMSD*_inactive_; *RMSD*_active_) tuples, following the same arched milestones distribution employed for ADRB1 (see Figure 3A and Figure S7). To deliver reliable statistics, each one-OPES simulation was carried out in triplicate. For clarity’s sake, the apo-MOR system sampled with the RPATH CV is referred to as *apo-MOR-OLD* hereafter, while the apo-MOR system sampled with the new EPATH CV as *apo-MOR-Euclidean*.

**Figure 3:**
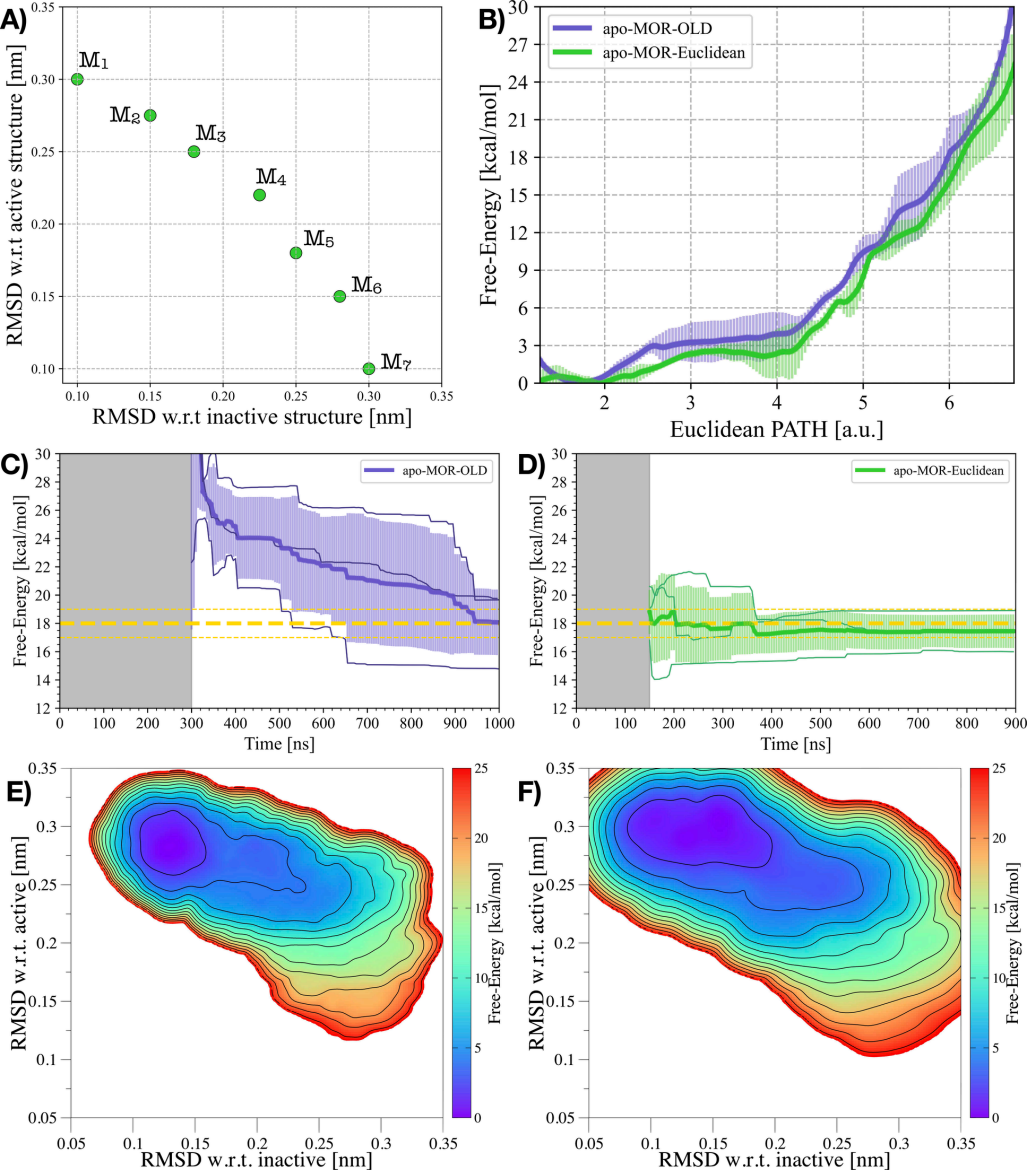
MOR’s activation using either the RPATH or the EPATH CVs. A) Milestones M_*i*_ of the EPATH CV upon which the *apo-MOR-OLD* and *apo-MOR-Euclidean* free energies have been reprojected. B) Comparison of the 1D FES as a function of the EPATH CV in (A) for the apo-*apo-MOR-OLD* and *apo-MOR-Euclidean* systems. C,D) Free-energy difference between inactive and active states in the *apo-MOR-OLD* (C) and *apo-MOR-Euclidean* (D) systems over time. In (B) and (C), the average free energy difference of *apo-MOR-OLD* is represented by a purple solid line, while the transparent purple area illustrates the standard deviation. Similarly, in (b) and (d) the average free energy difference of *apo-MOR-OLD* is represented by a green solid line, while the transparent green area illustrates the standard deviation. E,F) Average 2D FES concerning the RMSD of both the inactive and active structures for the *apo-MOR-OLD* (E) and *apo-MOR-Euclidean* (F) systems.

The free-energy results obtained from the two approaches are in excellent agreement, with both well capturing the thermodynamic equilibrium between inactive and active-like states. The agreement is evident from the one-dimensional free energy obtained by reweighting the accumulated bias potential of *apo-MOR-OLD* onto *apo-MOR-Euclidean*’s EPATH CV (see Figure 3B). However, the RPATH CV takes a rather long time to converge, with the free energy difference reaching an agreement between the independent replicas only at the end of the simulation (see Figure 3C). This indicates that, even though the CV itself went through an iterative optimization process, it is still far from ideal, and further refinements are necessary.

On the contrary, the EPATH CV once again converges faster than the RPATH, requiring a much shorter simulation time to achieve comparable estimates of the free-energy difference among the three independent replicas (see Figure 3D). Remarkably, no recursive CV optimization was needed to refine this PATH CV. In detail, for *apo-MOR-OLD*, the ΔG of activation is estimated to be 18.0 ± 1.0 kcal/mol after ∼800 ns, whereas *apo-MOR-Euclidean*’s OneOPES simulations converge to the ΔG value of ∼18.0 kcal/mol after only 200 ns. Similar considerations apply to the 2D FES measured on the (*RMSD*_inactive_; *RMSD*_active_) space: both sets of simulations are able to explore an analogous conformational space (see Figure 3E,F), with the *apo-MOR-Euclidean* trajectory being able to sample more thoroughly the fully active basin (*RMSD*_active_ < 0.1 nm; see Figure S7 for more details).

To further assess and compare the sampling quality of the two approaches, we additionally analyzed the rearrangement of key MOR microswitches along the activation coordinate. Specifically, we computed 2D FESs as a function of the activation pathway and structural descriptors associated with the *NPxxY*, *DRY*, and *YY* motifs. In all cases, the *apo-MOR-OLD* and *apo-MOR-Euclidean* simulations delivered similar landscapes, capturing both the qualitative ordering of microswitch transitions and the quantitative positioning of the free-energy minima (see Figure S8). We also calculated a 2D FES to characterize the hydration dynamics of the intracellular cavity in the proximity of the *YY* motif, a well-known hallmark of GPCR activation. This analysis reveals again a strong agreement between the two PATH CV definitions, with both approaches identifying analogous hydration patterns and their coupling to the receptor’s progression along the activation pathway (Figure S9). Finally, we examined the rearrangement of the MOR pseudoion lock,^60^ focusing on the residue pairs R^3.50^–T^6.34^ and V^3.54^–^L6.30^. Both approaches consistently capture an asynchronous disruption of these interactions along the activation pathway, with the loss of the V^3.54^–L^6.30^ hydrophobic contact occurring at early activation stages, followed by the breakage of the R^3.50^–T^6.34^ H-bond at later stages. This behavior, which is consistent with the progressive outward motion of TM6, is reproduced by both *apo-MOR-OLD* and *apo-MOR-Euclidean* simulations, further supporting the equivalence of the two PATH CV constructions (Figure S10).

## Advantages of the Euclidean PATH

To better understand how EPATH effectively drives GPCR activation, we analyzed the distribution of sampled configurations across the two collective variables, *s*(*M*) and *z*(*M*). In EPATH, the intermediate values of the *s*(*M*) coordinate correspond to conformations that are simultaneously distant from both the inactive and active references in the RMSD space. It is worth stressing that RMSD is a spherical yet anisotropic descriptor: a high RMSD value corresponds to a multitude of structurally distinct states equally distant from the reference state.^61^ For this reason, using RMSD alone as a CV in enhanced sampling is not particularly effective and may lead to poorly converged results.

In contrast, the EPATH CV couples two RMSD measures, thereby embedding directionality throughout its range in a push–pull fashion. This bivariate description enables detours away from the end states while avoiding the vast and uninformative region of high-RMSD configurations where simulations typically suffer from hysteresis. An important role in this context is played by the presence of loose restraints on the path deviation *z* and of coupled restraints between the progress CV *s* and microswitches *PIF* and *NPxxY*. This helps the convergence by aligning the conformation of the microswitches with the activation of the macroswitches. Another important element is the presence of the membrane that confines the GPCR and prevents it from exploring high-in-energy partially unfolded states.

Importantly, EPATH does not require a tedious definition of all of the intermediate milestones. Instead, in RPATH the definition of these milestones is crucial. The milestones are not known *a priori* and are typically extracted from steered MD trajectories. A suboptimal choice of the intermediate milestones decreases the efficiency of the method by forcing the sampling of conformations that are far from the RPATH. EPATH has fine resolution near the end points (where the corresponding structures and energy basins are well-defined), while remaining less well-defined in the middle, where the receptor naturally explores a broader ensemble. When combined with OneOPES, and auxiliary biases on the microswitches and water, this transition state region is crossed rapidly and reversibly, ensuring faster convergence than that with RPATH.

This behavior is clearly illustrated in Figure 4. In both *apo-MOR-OLD* (see Figure 4A) and *apo-MOR–Euclidean* (see Figure 4B), the intermediate portions of the EPATH are characterized by a plethora of slightly out-of-path frames (mostly between *s*(*M*) ∼ 4 and *s*(*M*) ∼ 5). Performing a cluster analysis on the trajectory frames confirms that the intermediate portion of the EPATH encompasses distinct structural families (see Figures S10 and S11 for further information). Notably, the simulation frames belonging to the first and last milestones (i.e., *M*_1_ and *M*_7_) can be grouped into very few cluster families, with the most probable ones containing more than 95% of the cluster populations. Conversely, the more we approach the central values of the EPATH, the more we can appreciate both an increase in cluster families and a decrease in their relative abundance.

**Figure 4:**
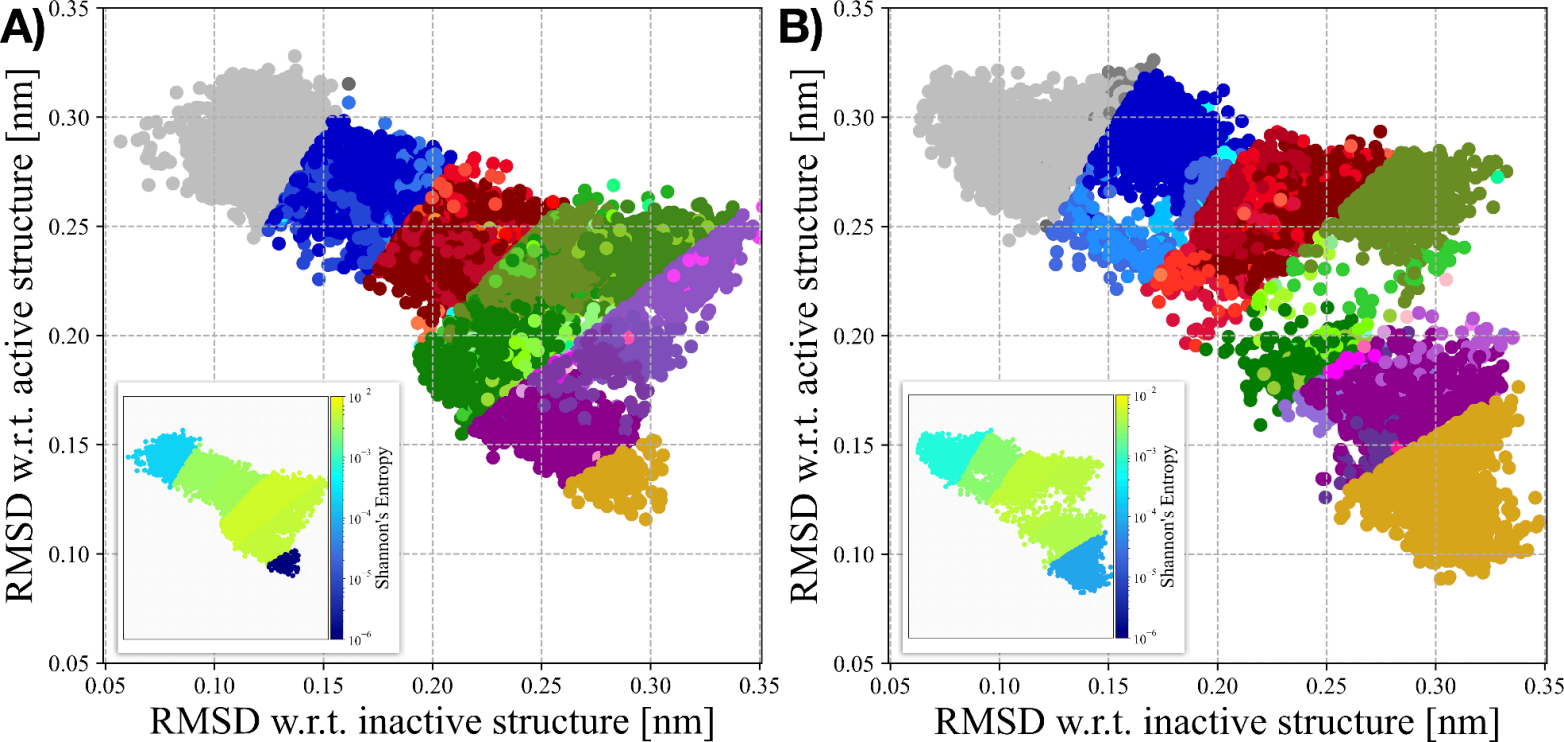
Analysis of the conformational heterogeneity observed in *apo-MOR-OLD* and *apo-MOR-Euclidean* OneOPES simulations. A,B) Sampling distributions of the *apo-MOR-OLD* (A) and *apo-MOR-Euclidean*. (B) OneOPES simulations projected onto the (*RMSD*_inactive_; *RMSD*_active_) space. Frames are grouped according to their Euclidean PATH interval (M1–M2, M2–M3, M3–M4, M4–M5, M5–M6, M6–M7), and points within each interval are colored according to the cluster they belong to (gray, blue, red, green, purple, and yellow palettes, respectively). In both panels, a small inset displays the same (*RMSD*_inactive_; *RMSD*_active_) sampling colored by the local Shannon entropy, providing a complementary view of the conformational heterogeneity within each region of the activation landscape.

To quantitatively characterize how the size of the conformational pool varies along the progress coordinate, we turned to information theory and computed the Shannon entropy associated with the cluster populations at each milestone (see the paragraph “Cluster Analysis and Shannon’s Entropy” for additional details). Consistent with our qualitative observations, both *apo-MOR-OLD* and *apo-MOR-Euclidean* exhibit the same global behavior: entropy reaches its minimum at the terminal milestones, where the conformational space is tightly focused around well-defined inactive and active structures and rises sharply toward the center of the path (see Figure 4A,B). Remarkably, in both formulations, the entropy in the *M*_4_–*M*_5_ interval increases by nearly 3 orders of magnitude relative to the end points, quantitatively confirming that the intermediate milestones host a vastly broader and more diverse ensemble of receptor conformations. This information-theoretic analysis reinforces the picture emerging from clustering and structural projections: the EPATH naturally captures the physical widening of the conformational landscape in the transition region, while maintaining strong definition at the two end points.

In conclusion, GPCRs are central regulators of cellular signaling and remain the targets of a large fraction of therapeutic compounds. A key determinant of their pharmacological action is the balance between inactive and active conformational states, which underlies the agonist–antagonist axis. Obtaining reliable free-energy differences between inactive and active reference states is therefore a pivotal step toward comparing receptor activation thermodynamics and assessing ligand effects. However, the inherent complexity of GPCR conformational landscapes poses major challenges for molecular simulations, requiring strategies that are accurate, practical, and transportable.^8,62^

In this work, we introduced a streamlined protocol for building PATH CVs within the OneOPES enhanced sampling framework. By eliminating the tedious trial-and-error procedure of the standard path definition, the new approach reduces the user effort required to set up simulations while maintaining the accuracy of the computed free-energy landscapes. Its application to two prototypical class A GPCRs,^63^ ADRB1 and MOR, demonstrates that this approach reproduces the thermodynamic results of the traditional method with a significantly faster convergence. An in-depth analysis of MOR further confirmed that the mechanistic details of activation, including side-chain microswitches and hydration changes, are faithfully captured. Additional analyses reported in the Supporting Information further show that alternative PATH definitions, including a straight Euclidean path directly connecting inactive and active reference states, lead to consistent but less efficient sampling, supporting the robustness of the proposed construction (see Figure S13).

An important question is whether providing additional information about the intermediate region of the activation landscape could further improve the approach. In our view, a balance must be struck between the ease of use and computational efficiency. For in-depth mechanistic studies of a single receptor, more elaborate CV refinement strategies may indeed offer advantages. However, when the goal is to analyze multiple GPCRs and to reliably estimate activation free energies without extensive system-specific optimization, the Euclidean PATH approach presented in this paper provides a robust, easy-to-build solution that delivers reliable sampling between end states.

Nonetheless, it is also important to acknowledge the limitations of RMSD-based metrics. As previously stated, at large RMSD values, the number of distinct conformations grows rapidly, making such regions difficult to sample and ineffective to bias. The role of these restraints and their impact on free-energy convergence is demonstrated in the Supporting Information through simulations that employ looser or absent microswitch restraints. Moreover, the real reaction coordinate involves both the position of the helices (macroswitches) tracked by the RMSD and the state of the microswitches, which is not directly included in the geometric path. The efficiency of the present approach stems largely from the constrained conformational space imposed on the intermediate states of the microswitches by mild restraining potentials. This restricts the exploration to the “relevant” microswitch states, thereby accelerating the convergence of the free energies. The role of these restraints and their impact on free-energy convergence is demonstrated in the Supporting Information through simulations that employ looser or absent microswitch restraints (see Figures S14 and S15). Systems that experience broader structural rearrangement embedded in a less constrained environment, e.g., a globular protein in water solution, may demand additional care, as the effective size of RMSD space increases and sampling may become a major challenge. Another point to bear in mind is that the estimated barriers under these conditions will not be as reliable as the difference between the end-point (inactive and active) states, while the shape of the 1D free energy profile is clearly affected by the definition of the path.

Overall, this method is more general and easier to use than previous proposals and therefore represents an important step toward routinely using enhanced sampling approaches to study the activation of class A GPCRs. Our approach enhances efficiency and accessibility, paving the way for future investigations that will quantify the effects of ligands or mutations on GPCR conformational equilibria, offering valuable insights for drug discovery and pharmacological design.

## Supplementary Material





## Data Availability

The OneOPES enhanced sampling simulation input files are available on GitHub at the link https://github.com/valeriorizzi/GPCR_Euclidean_PATH.git and on the PLUMED NEST repository https://www.plumed-nest.org/eggs/26/002/.^64^ The script requires PLUMED,^54,55^ version 2.8 or later. The enhanced sampling simulations are run with GROMACS 2023.^53^

## Notes

Additional figures, tables and computational details on the CV building strategy and on OneOPES enhanced sampling simulations (PDF)
Transparent Peer Review report available (PDF)

## References

[1] Rask-Andersen M., Almén M. S., Schiöth H. B. (2011). Trends in the exploitation of novel
drug targets. Nat. Rev. Drug Discovery.

[2] Katritch V., Cherezov V., Stevens R. C. (2013). Structure-function
of the G protein–coupled
receptor superfamily. Annual review of pharmacology
and toxicology.

[3] Sriram K., Insel P. A. (2018). G protein-coupled
receptors as targets for approved
drugs: how many targets and how many drugs?. Molecular pharmacology.

[4] Weis W. I., Kobilka B. K. (2018). The molecular basis of G protein–coupled receptor
activation. Annual review of biochemistry.

[5] Venkatakrishnan A., Deupi X., Lebon G., Tate C. G., Schertler G. F., Babu M. M. (2013). Molecular signatures
of G-protein-coupled receptors. Nature.

[6] Dror R. O., Green H. F., Valant C., Borhani D. W., Valcourt J. R., Pan A. C., Arlow D. H., Canals M., Lane J. R., Rahmani R. (2013). Structural
basis for modulation of a G-protein-coupled
receptor by allosteric drugs. Nature.

[7] Huang W., Manglik A., Venkatakrishnan A. J., Laeremans T., Feinberg E. N., Sanborn A. L., Kato H. E., Livingston K. E., Thorsen T. S., Kling R. C. (2015). Structural
insights
into *μ* -opioid receptor activation. Nature.

[8] Latorraca N. R., Venkatakrishnan A. J., Dror R. O. (2017). GPCR Dynamics: Structures in Motion. Chem. Rev..

[9] Lopez-Balastegui M., Stepniewski T. M., Kogut-Günthel M. M., Di Pizio A., Rosenkilde M. M., Mao J., Selent J. (2025). Relevance
of G protein-coupled
receptor (GPCR) dynamics for receptor activation, signalling bias
and allosteric modulation. Br. J. Pharmacol..

[10] De
Felice A., Aureli S., Limongelli V. (2021). Drug repurposing
on G protein-coupled receptors using a computational profiling approach. Frontiers in molecular biosciences.

[11] Aranda-García D., Stepniewski T. M., Torrens-Fontanals M., García-Recio A., Lopez-Balastegui M., Medel-Lacruz B., Morales-Pastor A., Peralta-García A., Dieguez-Eceolaza M., Sotillo-Nuñez D. (2025). Large scale investigation of GPCR molecular
dynamics data uncovers allosteric sites and lateral gateways. Nat. Commun..

[12] Calderón J. C., Ibrahim P., Gobbo D., Gervasio F. L., Clark T. (2023). Activation/Deactivation
Free-Energy Profiles for the *β*2-Adrenergic
Receptor: Ligand Modes of Action. J. Chem. Inf.
Model..

[13] Conflitti P., Lyman E., Sansom M. S. P., Hildebrand P. W., Gutiérrez-de Terán H., Carloni P., Ansell T. B., Yuan S., Barth P., Robinson A. S. (2025). Functional dynamics
of G protein-coupled receptors reveal new routes for drug discovery. Nat. Rev. Drug Discov.

[14] D’Amore V. M., Conflitti P., Marinelli L., Limongelli V. (2024). Minute-timescale
free-energy calculations reveal a pseudo-active state in the adenosine
A2A receptor activation mechanism. Chem..

[15] Provasi D., Artacho M. C., Negri A., Mobarec J. C., Filizola M. (2011). Ligand-induced
modulation of the free-energy landscape of G protein-coupled receptors
explored by adaptive biasing techniques. PLoS
Comput. Biol..

[16] Mattedi G., Acosta-Gutiérrez S., Clark T., Gervasio F. L. (2020). A combined
activation mechanism for the glucagon receptor. Proc. Natl. Acad. Sci. U. S. A..

[17] Aureli S., Rizzi V., Piasentin N., Gervasio F. L. (2025). Enhanced sampling
and tailored collective variables yield reproducible free energy landscapes
of beta-1 adrenergic receptor activation. J.
Chem. Theory Comput..

[18] Rodríguez-Espigares I., Torrens-Fontanals M., Tiemann J. K. S., Aranda-García D., Ramírez-Anguita J. M., Stepniewski T. M., Worp N., Varela-Rial A., Morales-Pastor A., Medel-Lacruz B. (2020). GPCRmd uncovers the
dynamics of the 3D-GPCRome. Nat. Methods.

[19] Mafi A., Kim S.-K., Goddard W. A. (2022). The mechanism
for ligand activation
of the GPCR–G protein complex. Proc.
Natl. Acad. Sci..

[20] Calderón J. C., Ibrahim P., Gobbo D., Gervasio F. L., Clark T. (2023). General Metadynamics
Protocol To Simulate Activation/Deactivation of Class A GPCRs: Proof
of Principle for the Serotonin Receptor. J.
Chem. Inf. Model..

[21] Saleh N., Saladino G., Gervasio F. L., Clark T. (2017). Investigating
allosteric
effects on the functional dynamics of b2-adrenergic ternary complexes
with enhanced-sampling simulations. Chemical
science.

[22] Maria-Solano M. A., Choi S. (2023). Dynamic allosteric
networks drive adenosine A1 receptor activation
and G-protein coupling. Biophys. J..

[23] Deganutti G., Pipitò L., Rujan R. M., Weizmann T., Griffin P., Ciancetta A., Moro S., Reynolds C. A. (2025). Hidden GPCR structural
transitions addressed by multiple walker supervised molecular dynamics
(mwSuMD). elife.

[24] Hénin J., Lelièvre T., Shirts M. R., Valsson O., Delemotte L. (2022). Enhanced Sampling
Methods for Molecular Dynamics Simulations [Article v1.0]. Living Journal of Computational Molecular Science.

[25] Laio A., Gervasio F. L. (2008). Metadynamics: a
method to simulate rare events and
reconstruct the free energy in biophysics, chemistry and material
science. Rep. Prog. Phys..

[26] Fröhlking T., Aureli S., Gervasio F. L. (2025). Learning
committor-consistent collective
variables: Transition pathways. Nature Computational
Science.

[27] Yuan X., Raniolo S., Limongelli V., Xu Y. (2018). The molecular mechanism
underlying ligand binding to the membrane-embedded site of a G-protein-coupled
receptor. J. Chem. Theory Comput..

[28] Rizzi V., Aureli S., Ansari N., Gervasio F. L. (2023). OneOPES, a combined
enhanced sampling method to rule them all. J.
Chem. Theory Comput..

[29] Branduardi D., Gervasio F. L., Parrinello M. (2007). From A to
B in free energy space. J. Chem. Phys..

[30] Marchi M., Ballone P. (1999). Adiabatic bias molecular
dynamics: a method to navigate
the conformational space of complex molecular systems. J. Chem. Phys..

[31] Park S., Schulten K. (2004). Calculating potentials of mean force from steered molecular
dynamics simulations. J. Chem. Phys..

[32] Sutto L., Gervasio F. L. (2013). Effects of oncogenic
mutations on the conformational
free-energy landscape of EGFR kinase. Proc.
Natl. Acad. Sci. U. S. A..

[33] Kuzmanic A., Sutto L., Saladino G., Nebreda A. R., Gervasio F. L., Orozco M. (2017). Changes in the free-energy landscape of p38*α* MAP kinase through its canonical activation and
binding events as studied by enhanced molecular dynamics simulations. eLife.

[34] Meral D., Provasi D., Filizola M. (2018). An efficient
strategy to estimate
thermodynamics and kinetics of G protein-coupled receptor activation
using metadynamics and maximum caliber. J. Chem.
Phys..

[35] Pietrucci F., Saitta A. M. (2015). Formamide reaction
network in gas phase and solution
via a unified theoretical approach: Toward a reconciliation of different
prebiotic scenarios. Proc. Natl. Acad. Sci.
U. S. A..

[36] Polino D., Parrinello M. (2019). Kinetics of Aqueous Media Reactions via Ab Initio Enhanced
Molecular Dynamics: The Case of Urea Decomposition. J. Phys. Chem. B.

[37] Das S., Raucci U., Neves R. P. P., Ramos M. J., Parrinello M. (2023). How and When
Does an Enzyme React? Unraveling *α*-Amylase
Catalytic Activity with Enhanced Sampling Techniques. ACS Catal..

[38] Das S., Raucci U., Neves R. P. P., Ramos M. J., Parrinello M. (2024). Correlating
enzymatic reactivity for different substrates using transferable data-driven
collective variables. Proc. Natl. Acad. Sci.
U. S. A..

[39] Xu X., Kaindl J., Clark M. J., Hübner H., Hirata K., Sunahara R. K., Gmeiner P., Kobilka B. K., Liu X. (2021). Binding pathway determines norepinephrine
selectivity for the human *β*1AR over *β*2AR. Cell Research.

[40] Vigneron S. F., Ohno S., Braz J., Kim J. Y., Kweon O. S., Webb C., Billesbølle C. B., Srinivasan K., Bhardwaj K., Irwin J. J. (2025). Docking
14 Million Virtual
Isoquinuclidines against the *μ* and *κ* Opioid Receptors Reveals Dual Antagonists–Inverse
Agonists with Reduced Withdrawal Effects. ACS
Central Science.

[41] Wang Y., Zhuang Y., DiBerto J. F., Zhou X. E., Schmitz G. P., Yuan Q., Jain M. K., Liu W., Melcher K., Jiang Y. (2023). Structures of the entire
human opioid receptor family. Cell.

[42] Invernizzi M., Parrinello M. (2022). Exploration
vs convergence speed in adaptive-bias enhanced
sampling. J. Chem. Theory Comput..

[43] Invernizzi M., Piaggi P. M., Parrinello M. (2020). Unified approach
to enhanced sampling. Physical Review X.

[44] Hauser A. S., Kooistra A. J., Munk C., Heydenreich F. M., Veprintsev D. B., Bouvier M., Babu M. M., Gloriam D. E. (2021). GPCR activation
mechanisms across classes and macro/microscales. Nature structural & molecular biology.

[45] Rizzi V., Bonati L., Ansari N., Parrinello M. (2021). The role of
water in host-guest interaction. Nat. Commun..

[46] Ansari N., Rizzi V., Parrinello M. (2022). Water regulates
the residence time
of Benzamidine in Trypsin. Nat. Commun..

[47] Karrenbrock M., Borsatto A., Rizzi V., Lukauskis D., Aureli S., Luigi Gervasio F. (2024). Absolute Binding
Free Energies with
OneOPES. J. Phys. Chem. Lett..

[48] Ding X., Aureli S., Vaithia A., Lavriha P., Schuster D., Khanppnavar B., Li X., Blum T. B., Picotti P., Gervasio F. L. (2024). Structural
basis of connexin-36 gap junction
channel inhibition. Cell Discovery.

[49] Febrer
Martinez P., Rizzi V., Aureli S., Gervasio F. L. (2024). Host–Guest
Binding Free Energies à la Carte: An Automated OneOPES Protocol. J. Chem. Theory Comput..

[50] Aureli S., Bellina F., Rizzi V., Gervasio F. L. (2024). Investigating
ligand-mediated
conformational dynamics of pre-miR21: A machine-learning-aided enhanced
sampling study. J. Chem. Inf. Model..

[51] Schulze M., Khakhula T., Piasentin N., Aureli S., Rizzi V., Gervasio F. L. (2025). All you need is
water: Converging ligand binding simulations
with hydration collective variables. J. Chem.
Phys..

[52] Grahl A., Abiko L. A., Isogai S., Sharpe T., Grzesiek S. (2020). A high-resolution
description of *β*1-adrenergic receptor functional
dynamics and allosteric coupling from backbone NMR. Nat. Commun..

[53] Abraham M. J., Murtola T., Schulz R., Páll S., Smith J. C., Hess B., Lindahl E. (2015). GROMACS: High
performance
molecular simulations through multi-level parallelism from laptops
to supercomputers. SoftwareX.

[54] Tribello G. A., Bonomi M., Branduardi D., Camilloni C., Bussi G. (2014). PLUMED 2: New feathers for an old
bird. Computer
physics communications.

[55] Tribello G. A., Bonomi M., Bussi G., Camilloni C., Armstrong B. I., Arsiccio A., Aureli S., Ballabio F., Bernetti M., Bonati L. (2025). PLUMED Tutorials: A collaborative,
community-driven learning ecosystem. J. Chem.
Phys..

[56] Abiko L. A., Grahl A., Grzesiek S. (2019). High pressure
shifts the *β*1-adrenergic receptor to the active
conformation
in the absence of G protein. J. Am. Chem. Soc..

[57] Su M., Paknejad N., Zhu L., Wang J., Do H. N., Miao Y., Liu W., Hite R. K., Huang X.-Y. (2022). Structures
of *β*1-adrenergic receptor in complex with Gs
and ligands of different efficacies. Nat. Commun..

[58] Volkow N. D., Blanco C. (2021). The changing opioid
crisis: development, challenges
and opportunities. Molecular psychiatry.

[59] Che T., Roth B. L. (2023). Molecular basis
of opioid receptor signaling. Cell.

[60] Huang P., Visiers I., Weinstein H., Liu-Chen L.-Y. (2002). The local environment
at the cytoplasmic end of TM6 of the *μ* opioid
receptor differs from those of rhodopsin and monoamine receptors:
introduction of an ionic lock between the cytoplasmic ends of helices
3 and 6 by a L6. 30 (275) E mutation inactivates the *μ* opioid receptor and reduces the constitutive activity of its T6.
34 (279) K mutant. Biochemistry.

[61] Kufareva, I. ; Abagyan, R. In Homology Modeling: Methods and Protocols; Orry, A. J. W. , Abagyan, R. , Eds.; Humana Press: Totowa, NJ, 2012; pp 231–257.

[62] Anantakrishnan S., Naganathan A. N. (2023). Thermodynamic architecture and conformational plasticity
of GPCRs. Nat. Commun..

[63] Marino K. A., Prada-Gracia D., Provasi D., Filizola M. (2016). Impact of
lipid composition
and receptor conformation on the spatio-temporal organization of *μ*-opioid receptors in a multi-component plasma membrane
model. PLoS computational biology.

[64] Bonomi M., Bussi G., Camilloni C., Tribello G. A. (2019). Promoting transparency
and reproducibility in enhanced molecular simulations. Nat. Methods.

