# Supplementary material for: A Transferable and Robust Computational Framework for Class A GPCR Activation Free Energies
Source: J Phys Chem Lett. 2026 Mar 3;17(10):2974–83. doi: 10.1021/acs.jpclett.5c03834 (PMC12990105; doi:10.1021/acs.jpclett.5c03834)
Supplement: Supplementary file 2 [file jz5c03834_si_002.pdf]

jz-2025-03834q.R1

Name: Peer Review Information for "A Transferable and Robust Computational Framework for Class A GPCR Activation Free Energies"

First Round of Reviewer Comments

Reviewer: 1

Comments to the Author

The authors build upon his previous work on development of enhanced sampling methods (OneOPES metadynamics) and optimized collective variables (path-based CV) for simulating the activation mechanism of GPCRs and present a new computational framework. In particular, they propose a new definition of the path-based CVs based not on a single RMSD, but an RMSD difference between active and inactive states. By applying this Euclidean definition of the path CV to run OneOPES simulations on two prototypical class A GPCRs, they are able to show that such approach is able to reproduce previous results while converging significantly faster, opening the way to its application to other class A GPCRs.

The manuscript is clearly written, but the authors might want to clarify some details and expand the discussion of the results, possibly in the supporting information due to the length limitations of the letter format. In particular, comparison with previous experimental and computational studies on the two receptors investigated in this work could help validate the results obtained here. Please see below for further details.

- Page 8: Can the authors provide more details of their choice of the slightly arched path above the diagonal used to connect the end states?

- Page 9: Can the authors elaborate on how application to a second GPCR (mu-opioid receptor) can show improved transferability? Though the mu-opioid receptor belongs to

another GPCR family (peptide receptors) compared to the beta1 adrenergic receptor (aminergic receptors), there is no discussion for either receptor regarding how the results presented here compare with other reports of the activation mechanism of these two receptors in the literature.

- Page 9: For the readers not familiar with GPCRs, the authors should spell out that the two structures mentioned here (PDB 7BVQ and 9MQJ) correspond to inactive states of the corresponding receptors (as the latter was solved in the presence of an antagonist). Moreover, the authors should explain which ADRB1 and MOR structures were used to define the respective active states. I could not find this information either in the main text or the supporting information.

- Page 10: The authors should provide further details on the parabolic restraints used for the PIF and NPxxY microswitches and explain why the other additional CVs based on microswitches DRY and YY did not require such restraints.

- Page 10: I would suggest making the OneOPES enhanced sampling simulation input files available not only on GitHub, but also on the PLUMED-NEST repository, for the benefit of the PLUMED user community.

- Page 11: Can the authors clarify if the Calphas used for the clustering are the same used for the RMSD definition? (i.e. set of Calpha atoms that make up the secondary structure of the receptor)

- Page 16: The authors mention that the qualitative ordering of the microswitch transitions and the quantitative position of the free energy minima are similar for both versions of the path-based CVs. Can the authors provide further details (possibly in the SI) regarding the mechanistic insights obtained for the activation mechanisms of ADRB1 and MOR? Moreover, is there experimental and/or computational data on these receptors that can be used to validate the activation mechanisms obtained? (e.g. experimental structures of the intermediate states or previous computational studies using other enhanced sampling methods)

- Page 17: As mentioned above, the authors should provide further details on the restraints used (possibly in the Methods). Even if they might have already been used in their previous work, the present manuscript should be self-contained.

- Page 20: Can the authors elaborate on potential applications of their workflow to multiple GPCRs? For instance, would it be useful to compare mutants or genetic variants of the same GPCR? or GPCRs belonging to the same family (e.g. ADRB1 and ADRB2)?

Reviewer: 2

Comments to the Author

This well-written manuscript presents a new strategy for constructing and employing PATH CVs, aiming to significantly reduce manual intervention while improving accuracy. This would be an excellent contribution, since the construction of a good reference PATH CV is extremely tedious, and the results are usually far from optimal. However, the authors suggest an arbitrary PATH CV based on a few tuples corresponding to the RMSDs to the inactive and active endpoint structures. I agree that the activation path is likely neither the diagonal nor one with low RMSDs to the reference states, but please elaborate on the choice of such milestones, the 'entropic nature' of the basin considered (as stated on page 8), as this important setup needs corroboration; otherwise, it appears too much arbitrary.

Another major concern I have is that, as stated in the Conclusion, 'A key determinant of their pharmacological action is the balance between inactive and active conformational states, which underlies the agonist–antagonist axis. Obtaining reliable free-energy profiles for these transitions is therefore a critical step toward understanding receptor function and quantifying the impact of ligand binding.' However, the work falls short of delivering mechanistic insights into the agonist/antagonist transition(s). According to NMR spectroscopy, apo class A GPCRs populate the active state at thermal equilibrium, albeit with much lower probability than the inactive state. This is not reflected in the FESs presented, which show the highest energy or a saddle near the active-state endpoint rather than a secondary minimum. This discrepancy is particularly evident for μ-OR (Figure 3B). Can the authors address this important aspect?

Additional concerns:

- The manuscript might be difficult to follow for readers who are not GPCR experts. Although the work focuses on structural transitions, no full GPCR structures are shown in the main text (only in the Supplementary Information). Including representative structures in the main figures would improve clarity and accessibility. For example, to show where the conserved motifs are located and explain the main differences between active and inactive states. Compare representative structures from minima to either the inactive or active states to provide structural insights into the activation pathway.

- Can the author propose the temporary sequence of motifs that switch along the activation, or some other novel structural insights? This would be potentially very valuable information in the field.

- The proposed approach appears tailored to class A GPCRs and may not be directly applicable to other GPCR classes. Please clarify this limitation explicitly.

SPECIFIC COMMENTS

INTRODUCTION

1. In addition to unbiased and enhanced MD simulations, adaptive MD methods should be mentioned, e.g., AdaptiveGoal: https://doi.org/10.1038/s41598-019-50752-6

2. Since GPCR activation typically occurs in concert with G protein interactions (full activation usually requires G protein binding), MD approaches that include intracellular effectors should be cited:

- MetaD: https://elifesciences.org/articles/90773, https://www.pnas.org/doi/10.1073/pnas.2110085119

- GaMD: https://www.pnas.org/doi/10.1073/pnas.2203702119

- SuMD: https://doi.org/10.7554/eLife.96513.4

METHODS

- System Preparation: The description accounts for inactive endpoints (e.g., μ-opioid 9MQJ structure) but lacks details on active states. From PLUMED files, PDB 7BTS was used as the active reference; please add the description of the active state structure preparation.

- E3.41 protonation: Please clarify that this is because it faces the membrane.

- Besides the equilibration protocol, please provide more details on MD production (ensemble, integration time step, etc.).

- Page 6: When discussing λ, specify that for RPATH CV, it should be based on the distance between reference structures. Is it the same here? How far apart should the RMSD tuples representing the arbitrary path be?

- Page 9: Please clarify about the RMSD alignment; does “90%” refer to all Cα atoms excluding loops, or another criterion? How was the efficiency of this generic choice assessed?

- Figure S2: Add motif names to panels for clarity.

- For motif bias, how were CUSTOM function parameters (e.g., 0.0045, 1.22) determined?

- I suggest merging the plots of the Ms with the 2D FES (e.g. Figure 2D with Figure S4A) to deliver the same data in one, more informative plot.

- Page 13: Can the authors specify the improvement in computational time due to the EPATH CV? How long does a simulation require to achieve convergence?

- Page 13: Please provide details on the steered MD protocol used to sample and extrapolate the RPATH CV, as well as the MetaD settings used (λ values, etc.).

Author's Response to Peer Review Comments:

Dear Journal of Physical Chemistry Letters Editor,
We appreciate the reviewers for their constructive feedback and insightful questions. We have revised and enhanced our original manuscript according to the suggestions received.

Below, we present each reviewer’s comment in black, followed by our corresponding answers in blue. Additionally, we have highlighted in red the changes made in the revised version of the manuscript.

Kind regards,

Francesco Luigi Gervasio, on behalf of all authors

Reviewer: 1

Recommendation: *This paper is publishable subject to minor revisions noted. Further review is not needed.*

The authors build upon his previous work on development of enhanced sampling methods (OneOPES metadynamics) and optimized collective variables (path-based CV) for simulating the activation mechanism of GPCRs and present a new computational framework. In particular, they propose a new definition of the path-based CVs based not on a single RMSD, but an RMSD difference between active and inactive states. By applying this Euclidean definition of the path CV to run OneOPES simulations on two prototypical class A GPCRs, they are able to show that such approach is able to reproduce previous results while converging significantly faster, opening the way to its application to other class A GPCRs.
We thank Reviewer #1 for the positive evaluation of our manuscript and for appreciating both the methodological innovation and the improved efficiency of the proposed sampling scheme.

The manuscript is clearly written, but the authors might want to clarify some details and expand the discussion of the results, possibly in the supporting information due to the length limitations of the letter format. In particular, comparison with previous experimental and computational studies on the two receptors investigated in this work could help validate the results obtained here. Please see below for further details.

- Page 8: Can the authors provide more details of their choice of the slightly arched path above the diagonal used to connect the end states?

This is indeed a relevant methodological point. Defining the path directly along the diagonal still results in reasonable free energy landscapes. However, this choice corresponds to a linear transformation of one crystal structure into another which does not map onto the 'real' path of minimal free energy (see panel E in the figure below), resulting in a worse sampling along the PATH collective variable and larger statistical uncertainties in the estimated freeenergy differences. The figure below makes this clear. It shows the results obtained for ADRB1 using a diagonal path definition in Fig. L-1. The arched path allows the system to explore conformations that are simultaneously "dissimilar" from both the inactive and active states in the chosen RMSD space. This enriched conformational pool better corresponds to the minimum free energy path connecting the inactive and active basins (and vice-versa), ultimately leading to improved convergence of the free-energy landscape.

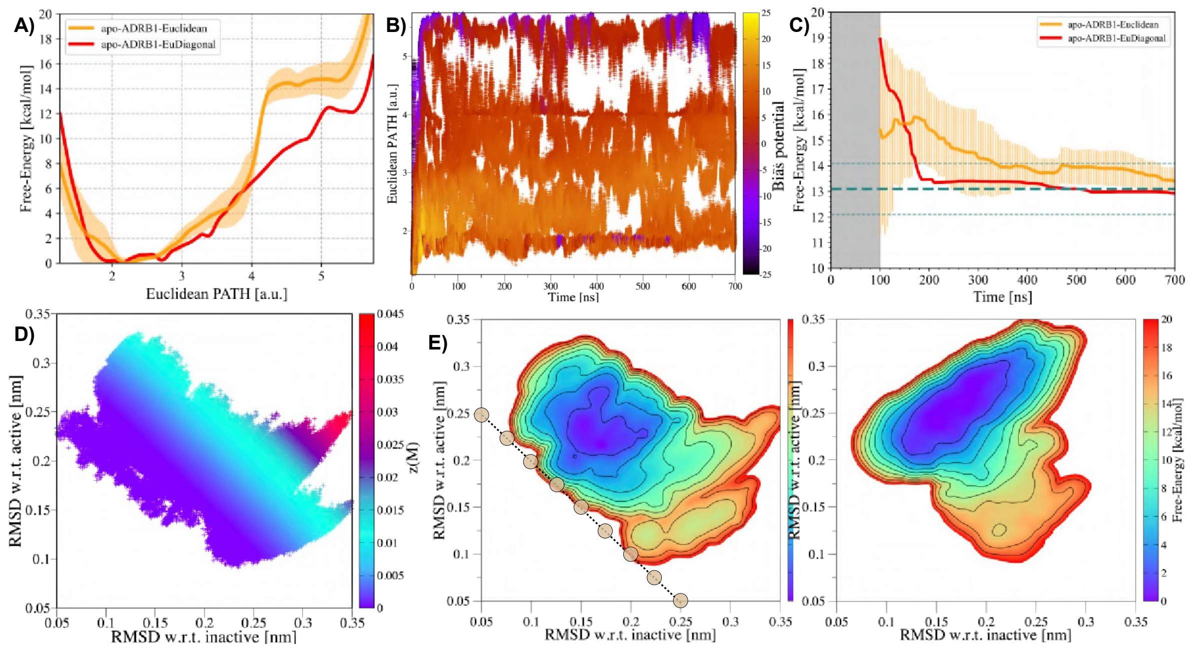

Figure L-1: **OneOPES simulation of apo-ADRB1 using an EPATH built along the diagonal connecting the inactive and active reference structures. a)** 1D FES of ADRB1 obtained from the diagonal Euclidean PATH, superimposed on the reference 1D FES reported in Fig. 2. **b)** Time evolution of the sampling along the EPATH, coloured by the accumulated bias potential. **c)** ΔΔG between the inactive and active states as a function of simulation time, compared with the corresponding result shown in Fig. 2. The reference value is indicated by the teal dashed line, which is from Aureli et al. [1]. **d)** Sampling projected onto the ($RMSD_{inactive}$; $RMSD_{active}$) space, coloured according to the Z(M) values (colorbar on the right side). **e)** Side-byside comparison of the 2D FES in the ($RMSD_{inactive}$; $RMSD_{active}$) space obtained from the present OneOPES simulation (left) and from the OneOPES simulations reported in Fig. 2 (right). The milestones composing the diagonal EPATH are shown through beige circles.

- Page 9: Can the authors elaborate on how application to a second GPCR (mu-opioid receptor) can show improved transferability? Though the mu-opioid receptor belongs to another GPCR family (peptide receptors) compared to the beta1 adrenergic receptor (aminergic receptors), there is no discussion for either receptor regarding how the results presented here compare with other reports of the activation mechanism of these two receptors in the literature.

We would like to emphasize that the present sampling strategy is intended to be applicable to class-A GPCRs, for which a common activation architecture and a common set of conserved microswitches is known [2]. Within this framework, we define transferability as the ability of the proposed CVs and sampling scheme to capture receptor-specific activation pathways while relying on the same general methodological setup. In this context, the application to the MOR demonstrates that the approach remains

effective even when applied to a receptor exhibiting distinct microscopic activation features. In particular, compared to our original work on ADRB1, MOR lacks the canonical ionic lock between $R^{3.50}$ and $E/D^{6.30}$ that is commonly observed in many aminergic receptors [5, 3]. Instead, MOR activation involves alternative residue couples, including a stabilizing interaction between $R^{3.50}$ and $T^{6.34}$, as well as a concerted outward displacement of TM6 facilitated by the hydrophobic residue $L^{6.30}$ (please see Fig. S10 of the supplementary material). Importantly, our simulations indicate that the Euclidean PATH CV (together with the extra CVs) can accommodate different activation mechanisms within class A GPCRs.

- Page 9: For the readers not familiar with GPCRs, the authors should spell out that the two structures mentioned here (PDB 7BVQ and 9MQJ) correspond to inactive states of the corresponding receptors (as the latter was solved in the presence of an antagonist). Moreover, the authors should explain which ADRB1 and MOR structures were used to define the respective active states. I could not find this information either in the main text or the supporting information.

In the revised text we added this information (previously only reported in SI) also to the "Methods" section of the main text.

- Page 10: The authors should provide further details on the parabolic restraints used for the PIF and NPxxY microswitches and explain why the other additional CVs based on microswitches DRY and YY did not require such restraints.

Using parabolic restraints on the selected microswitches (i.e. PIF and YY) is a fundamental part of the strategy, informed by extensive testing detailed in our previous paper [1]. Cryo-EM structures and NMR experiments have shown that the PIF and YY motifs assume specific conformations in the active state. However, we observed that the PIF and YY motifs tend to explore non-relevant conformations during enhanced sampling (e.g. long distances not observed in experimental active structures), which can hinder convergence. The application of weak parabolic restraints was therefore introduced to confine these microswitches within physically relevant ranges close to the end-points, without restricting the transitions between the inactive and active states. To further address this point, we have performed an additional OneOPES simulation on ADRB1 in which the parabolic restraints on the microswitch-based CVs were weakened or removed (see Fig. L-2). The results of this test are shown below. When the parabolic restraint are weaker, the convergence is slower but still attained within approx. 400ns. Without parabolic restraints the relevant active conformation (helix out and YY motif engaged) is visited only once in 700ns, which significantly impacts the convergence. These important considerations are now reported and discussed in the revised Supplementary Information.

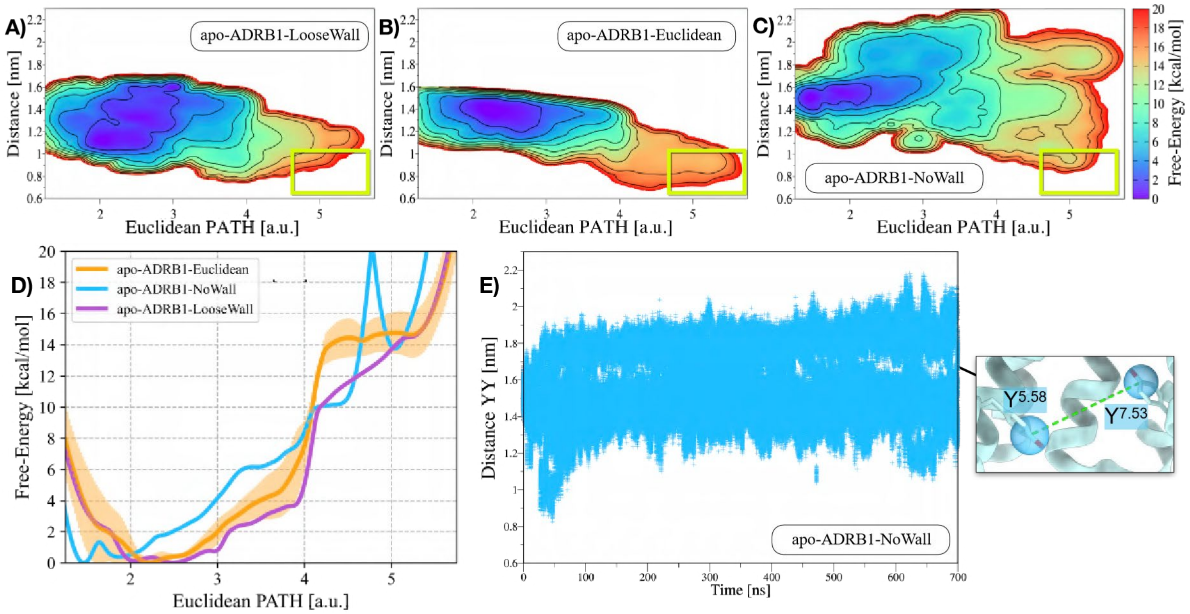

Figure L-2: **Outcomes of ADRB1 OneOPES simulations employing the Euclidean PATH under different microswitch restraint conditions. a-c)** 2D FES of the YYmotif as a function of the Euclidean PATH CV obtained from the *apo-ADRB1LooseWall* simulation (**A**), the reference OneOPES simulation reported in Fig. 2
(**B**), and the *apo-ADRB1-NoWall* simulation (**C**). The yellow rectangle highlights the EPATH region where the two tyrosines of the YY-motif are expected to approach each other to support ADRB1 activation. **d)** 1D FES of ADRB1 reported in Fig. 2, superimposed with the profiles obtained from the *apo-ADRB1-NoWall* and *apo-ADRB1LooseWall* OneOPES simulations. **e)** Time evolution of the YY-motif distance during the *apo-ADRB1-NoWall* simulation, showing that only one complete transition between inactive and active-like configurations is observed at the very beginning of the OneOPES simulation.

- Page 10: I would suggest making the OneOPES enhanced sampling simulation input files available not only on GitHub, but also on the PLUMED-NEST repository, for the benefit of the PLUMED user community.

We welcome the suggestion of Reviewer #1. We were planning to add the simulation input files to the PLUMED-NEST repository upon publication. We have created a new entry at https://www.plumed-nest.org/eggs/26/002/ and added its reference to the revised main text.

- Page 11: Can the authors clarify if the Calphas used for the clustering are the same used for the RMSD definition? (i.e., set of Calpha atoms that make up the secondary structure of the receptor).

Yes, the Cαs on which we have carried out the cluster analysis are the same upon which we calculated $RMSD_{inactive}$ and $RMSD_{active}$. We added this point to the "Methods" section.

- Page 16: The authors mention that the qualitative ordering of the microswitch transitions and the quantitative position of the free energy minima are similar for both versions of the path-based CVs. Can the authors provide further details (possibly in the SI) regarding the mechanistic insights obtained for the activation mechanisms of ADRB1 and MOR? Moreover, is there experimental and/or computational data on these receptors that can be used to validate the activation mechanisms obtained? (e.g., experimental structures of the intermediate states or previous computational studies using other enhanced sampling methods)

In the revised manuscript, we have expanded the discussion of the mechanistic insights obtained for ADRB1 and MOR, including the qualitative ordering of microswitch transitions and the positioning of the free-energy minima obtained with the two path-based CV definitions. Due to space limitations, a more detailed analysis has been added to the Supplementary Information (see Fig. S10), where we analyzed the behaviour of $R^{3.50}$-$T^{6.34}$ distance, which takes the place of the canonical ion lock in MOR [3]. We believe this additional discussion provides further validation of our results.

- Page 17: As mentioned above, the authors should provide further details on the restraints used (possibly in the Methods). Even if they might have already been used in their previous work, the present manuscript should be self-contained.

Due to the limits on the letter format's length, we have added a small analysis on the restraints used for the microswitches' distances in the Supplementary Information (see section "Supplementary Data 9").

- Page 20: Can the authors elaborate on potential applications of their workflow to multiple GPCRs? For instance, would it be useful to compare mutants or genetic variants of the same GPCR? or GPCRs belonging to the same family (e.g., ADRB1 and ADRB2)?

The proposed workflow is designed to be applicable to multiple class A GPCRs within a unified methodological framework, making it particularly suitable for comparative studies across related receptors or receptor variants. For instance, it can be used to compare closely related GPCRs within the same family (e.g., ADRB1 and ADRB2), as well as to assess the impact of point mutations or genetic variants on the activation free-energy landscape and microswitch rearrangements. To further illustrate this transferability, and trusting in the Reviewer's goodwill, we provide as additional material an activation profile of the class-A GPCR CCR5, obtained using the same workflow, in the presence of both an agonist and an antagonist (see Fig.L-3) CCR5 represents a more divergent case, as it lacks the canonical ionic lock and features a variant of the PIF microswitch (PIY), further distinguishing it from ADRB1. Despite these differences, the proposed approach remains able to capture a coherent activation mechanism. These results are provided for evaluation purposes and will be the subject of a separate dedicated study.

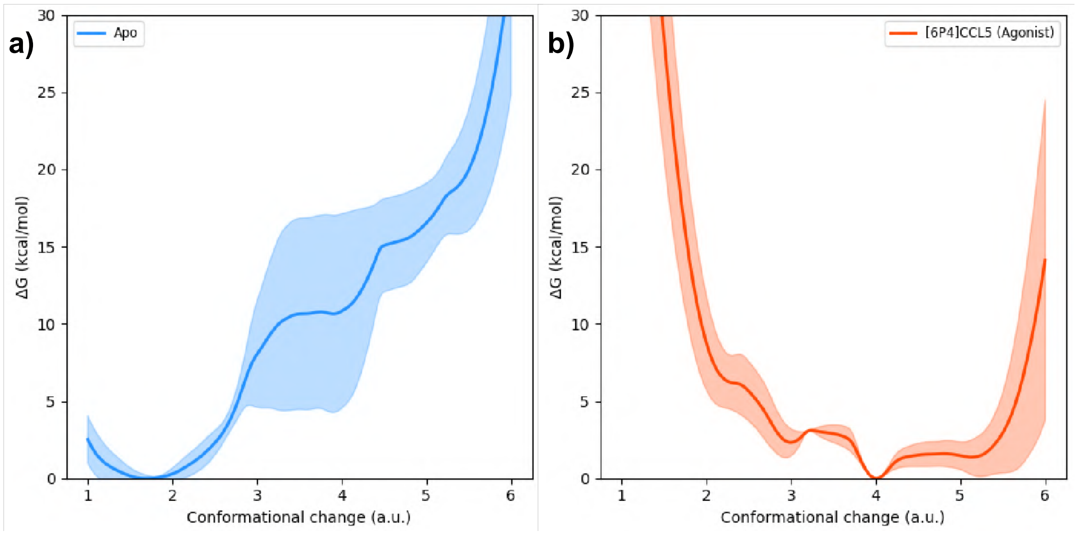

Figure L-3: **Free-energy profiles of CCR5 activation from OneOPES simulations.** 1D free-energy profile of **a)** apo-CCR5 and **b)** agonist-bound CCR5. The solid lines and the transparent areas are the average and the standard deviation of three independent OneOPES simulations, respectively.

Reviewer: 2

Recommendation: *This paper may be publishable, but major revision is needed; I would like to be invited to review any future revision.*

This well-written manuscript presents a new strategy for constructing and employing PATH CVs, aiming to significantly reduce manual intervention while improving accuracy. This would be an excellent contribution, since the construction of a good reference PATH CV is extremely tedious, and the results are usually far from optimal. However, the authors suggest an arbitrary PATH CV based on a few tuples corresponding to the RMSDs to the inactive and active endpoint structures. I agree that the activation path is likely neither the diagonal nor one with low RMSDs to the reference states, but please elaborate on the choice of such milestones, the 'entropic nature' of the basin considered (as stated on page 8), as this important setup needs corroboration; otherwise, it appears too much arbitrary.

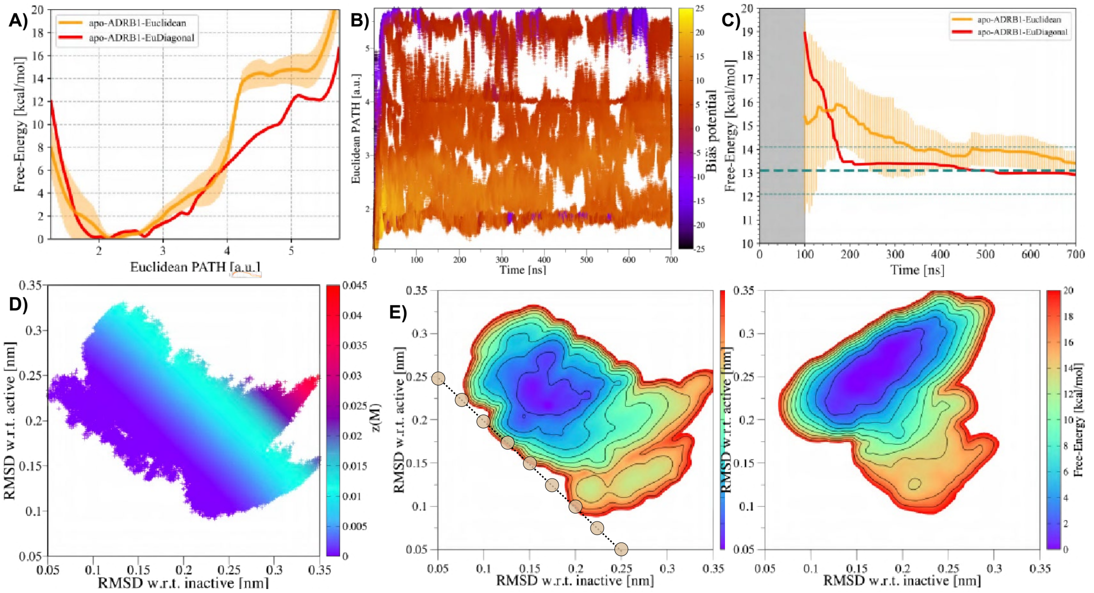

Figure L-4: **OneOPES simulation of apo-ADRB1 using an EPATH built along the diagonal connecting the inactive and active reference structures. a)** 1D FES of ADRB1 obtained from the diagonal Euclidean PATH, superimposed on the reference 1D FES reported in Fig. 2. **b)** Time evolution of the sampling along the EPATH, coloured by the accumulated bias potential. **c)** ΔΔG between the inactive and active states as a function of simulation time, compared with the corresponding result shown in Fig. 2. The reference value is indicated by the teal dashed line, which is from Aureli et al. [1]. **d)** Sampling projected onto the ($RMSD_{inactive}$; $RMSD_{active}$) space, coloured according to the Z(M) values (colorbar on the right side). **e)** Side-byside comparison of the 2D FES in the ($RMSD_{inactive}$; $RMSD_{active}$) space obtained from the present OneOPES simulation (left) and from the OneOPES simulations reported in Fig. 2 (right). The milestones composing the diagonal EPATH are shown through beige circles.

As also discussed above in the reply to Rev. #1, this is indeed an important point. The proposed path defines a low-dimensional coordinate that facilitates enhanced sampling of the activation free-energy landscape. A straight path connecting the experimentally determined active and inactive states would correspond to “morphing” one state into another. However, this does not reflect the actual mechanism, which involves intermediate states that are not located along a line connecting the two states. To clarify this point, we performed an additional OneOPES simulation on ADRB1 in which the Euclidean PATH CV was built directly along the diagonal connecting the inactive and active reference states (see Fig. L-4). This alternative definition still delivers a decent agreement with the calculated free-energy difference, but it is less effective (see L-4B). As displayed in Fig. L-4D-E, even with a straight reference path, the system spontaneously explored an off-diagonal area of the ($RMSD_{inactive}$; $RMSD_{active}$) space leading to an arched path similar to those shown in Fig. 2. This straight path leads to the undersampling of the area ($RMSD_{inactive}$

~0.25; $RMSD_{active}$ ~0.32). This, in turn, results in an underestimation of the inactive-state basin. These results are now included and discussed in the Supplementary Information (see “Supplementary Data 8”).

Another major concern I have is that, as stated in the Conclusion, ‘A key determinant of their pharmacological action is the balance between inactive and active conformational states, which underlies the agonist–antagonist axis. Obtaining reliable free-energy profiles for these transitions is therefore a critical step toward understanding receptor function and quantifying the impact of ligand binding.’ However, the work falls short of delivering mechanistic insights into the agonist/antagonist transition(s). According to NMR spectroscopy, apo class A GPCRs populate the active state at thermal equilibrium, albeit with much lower probability than the inactive state. This is not reflected in the FESs presented, which show the highest energy or a saddle near the active-state endpoint rather than a secondary minimum. This discrepancy is particularly evident for μ-OR (Figure 3B). Can the authors address this important aspect?
There are two considerations related to this important aspect. The main is that all simulations were performed on apo (unliganded) receptors in the absence of G-protein or stabilizing intracellular partners. Under these conditions, the fully active state is not expected to be populated and unstable or only marginally stable. Recent high-resolution NMR measurements on ADRB1 of Wu et al. [6], indicate that even in the presence of an agonist, the active population is very limited since an agonist and the G protein are needed for activation. The same study reports exchange times corresponding to activation barriers of at least ~17 kcal/mol. The higher free energy observed near the active endpoint for MOR therefore reflects the observed experimental stability of the fully active conformation, rather than the absence of active-like states. The other important point to consider with respect to the shape of the 1D curve (lack of a nicely defined secondary minimum) is the specific free energy projection that we chose. The advantage of our 1D RMSD-based variable is that it is easy to define for class A GPCRs and captures the main features of the macroswitches. However it does not directly capture the state of the microswitches. This is important to bear in mind, considering that the experimental evidence in solution (NMR) shows that both the “pre-active state” which is populated in the presence of agonists but in the absence of a G-protein and the fully active state corresponds states in which the microswitches are in active conformations. In other words the real reaction coordinate combines the state of the microswitches as well as that of the macroswitches (the position of the helices). Thus it is the simplified nature of the chosen geometric path-like variable that results in a projection whereby the active state looks like a shoulder. In this regard, the 2D profiles (e.g. path vs. YY motif) are more effective at identifying the actual minima. Overall we agree with the reviewer that this is an important point and we have clarified the limits of the 1D projection in the revised manuscript and slightly tempered the

wording in the Conclusions. We now explicitly state that the present approach is designed to provide reliable estimates of relative free energies between inactive and active **reference** states and a consistent description of activation pathways. For a quantitative characterization of the equilibrium distribution of inactive, pre-active and active sub-states the microswitches have to be considered so we advise using multidimensional projections of the FES.

Additional concerns:

- The manuscript might be difficult to follow for readers who are not GPCR experts. Although the work focuses on structural transitions, no full GPCR structures are shown in the main text (only in the Supplementary Information). Including representative structures in the main figures would improve clarity and accessibility. For example, to show where the conserved motifs are located and explain the main differences between active and inactive states. Compare representative structures from minima to either the inactive or active states to provide structural insights into the activation pathway.

We note that representative structural views highlighting the orientation of the conserved microswitches were already included in the original Supplementary Information (e.g., Figs. S2, S5, S8, and S9). Nevertheless, to improve clarity and accessibility for non-specialist readers, we have expanded Fig. 1 in the main text to provide a clearer overview of class A GPCR architecture and to explicitly indicate the conserved microswitches that are accelerated within our sampling strategy. We believe that this revised figure, together with the detailed structural representations already provided in the Supplementary Information, significantly improves the readability and structural interpretation of the manuscript.

- Can the author propose the temporary sequence of motifs that switch along the activation, or some other novel structural insights? This would be potentially very valuable information in the field.

We agree that identifying the temporal sequence of microswitch rearrangements during GPCR activation would be highly valuable. However, we note that within replica-exchange–based enhanced sampling frameworks, such as OneOPES, the notion of a true temporal ordering of events is not well defined, as the dynamics are intentionally altered to enhance exploration of configurational space. Rather than providing a kinetic sequence, our approach allows us to characterize how conserved microswitches reorganize along the activation path defined by the collective variables. In the revised Supplementary Information, we have therefore added an analysis describing the progression of microswitch rearrangements as a function of the activation coordinate, highlighting consistent structural trends and intermediate features observed during the transition (see Fig. S10).

- The proposed approach appears tailored to class A GPCRs and may not be directly applicable to other GPCR classes. Please clarify this limitation explicitly.

We thank the Reviewer for this comment. We agree that the proposed approach is specifically designed for class A GPCRs, where a conserved activation architecture and well-characterized microswitches are present. We have now explicitly clarified this limitation in the “Introduction” and “Conclusions” sections of the revised manuscript.

SPECIFIC COMMENTS

INTRODUCTION

-In addition to unbiased and enhanced MD simulations, adaptive MD methods should be mentioned, e.g., AdaptiveGoal: https://doi.org/10.1038/s41598-019-50752-6

-Since GPCR activation typically occurs in concert with G protein interactions (full activation usually requires G protein binding), MD approaches that include intracellular effectors should be cited:

- MetaD: https://elifesciences.org/articles/90773, https://www.pnas.org/doi/10.1073/pnas.2110085119;
- GaMD: https://www.pnas.org/doi/10.1073/pnas.2203702119
- SuMD: https://doi.org/10.7554/eLife.96513.4

All of them have been included in the new version of the “Introduction” section of the revised manuscript.

METHODS

-System Preparation: The description accounts for inactive endpoints (e.g., μ-opioid 9MQJ structure) but lacks details on active states. From PLUMED files, PDB 7BTS was used as the active reference; please add the description of the active state structure preparation.

For the μ-opioid receptor, the active reference structure is described in the Supplementary Information (Supplementary Data 5, PDB ID: 8F7R). In addition, we have now explicitly added the description of the reference active structures used for both ADRB1 and MOR in the Methods section of the revised manuscript.

-E3.41 protonation: Please clarify that this is because it faces the membrane.

We added this information in the new “Supplementary Data 1” section of the revised Supplementary Information.

-Besides the equilibration protocol, please provide more details on MD production (ensemble, integration time step, etc.).

We added additional details in the revised Supplementary information.

-Page 6: When discussing λ, specify that for RPATH CV, it should be based on the distance between reference structures. Is it the same here? How far apart should the RMSD tuples representing the arbitrary path be?
In the present implementation, λ has the same role as in the original RPATH formulation: it controls the overlap between neighbouring milestones along the path. It should therefore be chosen so that consecutive reference points exhibit sufficient overlap, while avoiding significant overlap with distant ones. Although the Euclidean PATH is defined using tuples of RMSD values rather than single structural distances, the tuning principle remains unchanged. λ is selected relative to the spacing between adjacent reference tuples in RMSD space, and the same practical guidelines used in standard path CV implementations apply here.

-Page 9: Please clarify about the RMSD alignment; does "90%" refer to all Cα atoms excluding loops, or another criterion? How was the efficiency of this generic choice assessed? As displayed in Fig. L-5, we calculated the RMSD on the Cαs of the residues in red, while the amino acids in blue (belonging to ICL1, ECL1, and ECL3) have not been considered. In total, we measured the RMSD on 274 residues out of 288 (~ 95%). The choice of excluding ICL1, ECL1, and ECL3 was quite straightforward, due to their minor relevance in the activation of class-A GPCRs.

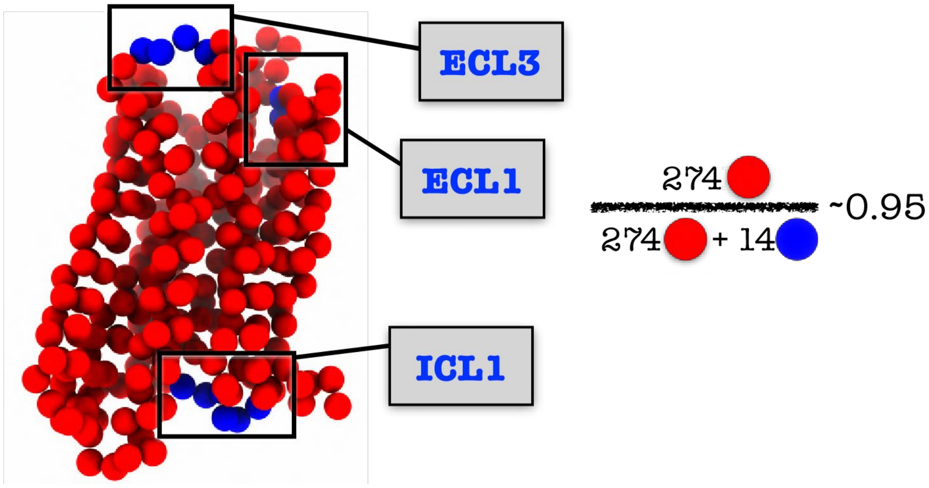

Figure L-5: **Residue selection for RMSD calculations of MOR.** The RMSD was computed on the Cα atoms of the residues highlighted in red, whereas residues shown in blue (ICL1, ECL1, and ECL3) were excluded from the calculation due to their limited role in class-A GPCR activation. In total, 274 out of 288 residues (~95%) were considered.

-Figure S2: Add motif names to panels for clarity.
We included them in the revised Supplementary information as part of Fig. S2.

-For motif bias, how were CUSTOM function parameters (e.g., 0.0045, 1.22) determined? So far, the parameters ruling the parabolic functions have been selected through trial-anderror, forcing the microswitches to approach the distances they should have in the reference active state without adding an external potential restraint that could limit the sampling of the active ensemble. These parabolic functions are crucial to achieve a rapid convergence and a high accuracy of the OneOPES simulations (see also reply to Rev. #1). To better highlight this point, we carried out additional simulations with looser CUSTOM function parameters and without parabolic restraints at all (see Fig. L-6). These results have been discussed in the revised Supplementary Information, in the section "Supplementary Data 9".

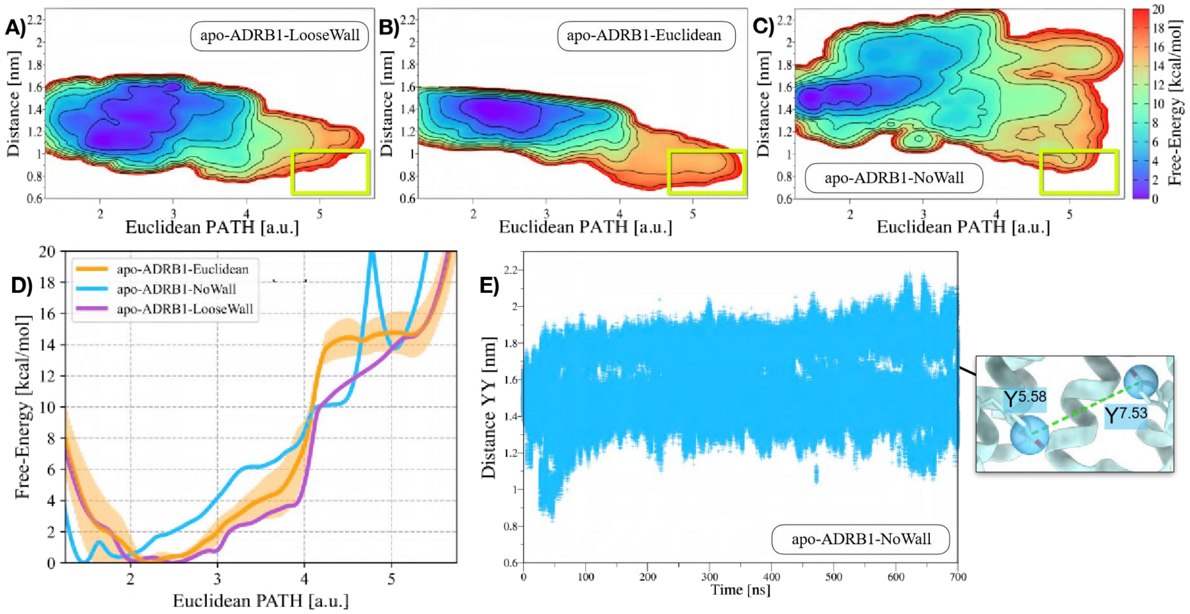

Figure L-6: **Outcomes of ADRB1 OneOPES simulations employing the Euclidean PATH under different microswitch restraint conditions. a-c)** 2D FES of the YYmotif as a function of the Euclidean PATH CV obtained from the *apo-ADRB1LooseWall* simulation (**A**), the reference OneOPES simulation reported in Fig. 2
(**B**), and the *apo-ADRB1-NoWall* simulation (**C**). The yellow rectangle highlights the EPATH region where the two tyrosines of the YY-motif are expected to approach each other to support ADRB1 activation. **d)** 1D FES of ADRB1 reported in Fig. 2, superimposed with the profiles obtained from the *apo-ADRB1-NoWall* and *apo-ADRB1LooseWall* OneOPES simulations. **e)** Time evolution of the YY-motif distance during the *apo-ADRB1-NoWall* simulation, showing that only one complete transition between inactive and active-like configurations is observed at the very beginning of the OneOPES simulation.

-I suggest merging the plots of the Ms with the 2D FES (e.g., Figure 2D with Figure S4A) to deliver the same data in one, more informative plot.

We thank Reviewer #2 for this constructive suggestion. We considered merging the milestone positions with the corresponding 2D FES plots; however, we found that overlaying these elements significantly reduced the readability of the free-energy landscapes, particularly given the number of milestones involved. For clarity, we therefore chose to keep the milestone information and the 2D FES representations in separate panels.

-Page 13: Can the authors specify the improvement in computational time due to the EPATH CV? How long does a simulation require to achieve convergence?
To have a clear comparison between the old path and the new euclidean path, we can compare the speed (in terms of ns/day) of the triplicates we run for the apo ADRB1 system in our previous work [1] with those in this new work. These are the same exact simulation boxes with roughly 125k atoms. For the old path (i.e., RPATH), we obtained an average speed of 83.3 ± 3.9 ns/day, while with the new one an average of 90.8 ± 1.7 ns/day, that is, a speed up of ∼ 10%. We recognize that performance depends largely on the systems size and the hardware used to run the simulations. Notably, we run these simulations on the cutting-edge Tier-0 infrastructures at the Swiss National Supercomputing Centre (CSCS), where each node consists of 4 Grace-Hopper chipsets, effectively splitting the eight communicating replicas within a single OneOPES run on 4 GPUs and 288 cores of a single node. Nevertheless, it should also be noted that in the Euclidean path formalism the cost of computing the distance is always nearly the same independently from then number of milestones, as only two RMSD distances need to be calculated. For the previous RPATH, instead, the cost will increase linearly with the number of milestones.

-Page 13: Please provide details on the steered MD protocol used to sample and extrapolate the RPATH CV, as well as the MetaD settings used (λ values, etc.).
We thank Reviewer #2 for having provided us the opportunity to clarify a few aspects of our work. We did not employ *MetaD* to carry out the steered MD. Instead, we performed the steered MD on the MOR GPCR by employing the dedicated *MOVINGRESTRAINT* bias in the PLUMED plugin. Selected Cαs of the GPCR are pushed towards a reference structure (in this case, PDB ID: 8F7R [4]), by employing the following harmonic bias:

$$V(s,t) = \frac{1}{2}k \cdot (s(t) - s_0)^2$$

where k is the elastic constant, $s(t)$ the RMSD value of the GPCR with respect to the structure 8F7R, and s_0 the reference value. For the steered MD on MOR, we employed a k value of 5000 $kJ \cdot mol^{-1} \cdot K^{-1} \cdot nm^{-2}$ for 2 ns, followed by a k value of 50000 $kJ \cdot mol^{-1} \cdot K^{-1} \cdot nm^{-2}$ for 18ns. Once again, we calculated MOR's RMSD on all Cα atoms excluding the ICL1 and ECL1 loops.

References

[1] Simone Aureli, Valerio Rizzi, Nicola Piasentin, and Francesco Luigi Gervasio. Enhanced sampling and tailored collective variables yield reproducible free energy landscapes of beta-1 adrenergic receptor activation. *Journal of Chemical Theory and Computation*, 21(15):7687–7700, 2025.

[2] Alexander S Hauser, Albert J Kooistra, Christian Munk, Franziska M Heydenreich, Dmitry B Veprintsev, Michel Bouvier, M Madan Babu, and David E Gloriam. Gpcr activation mechanisms across classes and macro/microscales. *Nature structural & molecular biology*, 28(11):879–888, 2021.

[3] Peng Huang, Irache Visiers, Harel Weinstein, and Lee-Yuan Liu-Chen. The local environment at the cytoplasmic end of tm6 of the μ opioid receptor differs from those of rhodopsin and monoamine receptors: introduction of an ionic lock between the cytoplasmic ends of helices 3 and 6 by a l6. 30 (275) e mutation inactivates the μ opioid receptor and reduces the constitutive activity of its t6. 34 (279) k mutant. *Biochemistry*, 41(40):11972–11980, 2002.

[4] Yue Wang, Youwen Zhuang, Jeffrey F DiBerto, X Edward Zhou, Gavin P Schmitz, Qingning Yuan, Manish K Jain, Weiyi Liu, Karsten Melcher, Yi Jiang, et al. Structures of the entire human opioid receptor family. *Cell*, 186(2):413–427, 2023.

[5] William I Weis and Brian K Kobilka. The molecular basis of g protein–coupled receptor activation. *Annual review of biochemistry*, 87(1):897–919, 2018.

[6] Feng-Jie Wu, Pascal S. Rieder, Layara Akemi Abiko, Anne Grahl, Daniel H ̈aussinger, and Stephan Grzesiek. Activation dynamics traced through a G protein–coupled receptor by 81 1H-15N NMR probes. *Science*, 388(6748):eadq9106, May 2025. Publisher: American Association for the Advancement of Science.

jz-2025-03834q.R2

Name: Peer Review Information for "A Transferable and Robust Computational Framework for Class A GPCR Activation Free Energies"

Second Round of Reviewer Comments

Reviewer: 2

Comments to the Author

The authors have addressed my concerns.

Reviewer: 1

Comments to the Author

The authors have satisfactorily addressed my comments.

Author's Response to Peer Review Comments:

Dear Editor,

Thank you for the prompt revision of our manuscript titled "A Transferable and Robust Computational Framework for Class A GPCR Activation Free Energies."

As requested, we have uploaded a further revised version of the manuscript, from which we have removed the section headings.

Please let us know if there are any additional issues.

Kind regards,

Francesco Luigi Gervasio, on behalf of all the authors.
